# Novel Aptamers Targeting Sclerostin Loop3 Improve Skeletal and Muscle Properties Without Adverse Cardiovascular Effects in Orchiectomized Mice

**DOI:** 10.1002/jcsm.13831

**Published:** 2025-06-04

**Authors:** Bingna Zhou, Jing Hu, Yuanyuan Yu, Lei Sun, Yanye Wang, Qian Zhang, Yan Jiang, Ou Wang, Xiaoping Xing, Weibo Xia, Luyao Wang, Ge Zhang, Mei Li

**Affiliations:** ^1^ Department of Endocrinology, Key Laboratory of Endocrinology, National Health and Family Planning Commission, Peking Union Medical College Hospital Chinese Academy of Medical Sciences and Peking Union Medical College Beijing China; ^2^ Law Sau Fai Institute for Advancing Translational Medicine in Bone and Joint Diseases (TMBJ), School of Chinese Medicine Hong Kong Baptist University Hong Kong SAR China

**Keywords:** adverse cardiovascular effects, aptamers, male osteoporosis, sarcopenia, sclerostin loop3

## Abstract

**Background:**

The Wnt/β‐catenin pathway and its bone‐specific inhibitor, sclerostin, play important roles in skeletal development and homeostasis. The humanized sclerostin antibody, romosozumab, can significantly increase bone mineral density (BMD) of patients with osteoporosis, but it may also increase cardiovascular adverse events, particularly in male patients. We try to investigate the effects of novel aptamers targeting the sclerostin loop3 on the skeleton and muscle of orchiectomized (ORX) mice.

**Methods:**

After 12 weeks of ORX surgery, mice were randomly assigned to receive treatment with sclerostin aptamers (Apc001OA or Apc001OA‐d6), alendronate (ALN), teriparatide (PTH 1–34) or phosphate‐buffered saline (PBS). After 12 weeks of treatment, skeletal and muscle properties and safety indicators were evaluated in detail.

**Results:**

Treatment with Apc001OA and Apc001OA‐d6 significantly increased trabecular BMD at the femur by +11.9% and +17.1%, improved parameters of bone microarchitecture (BV/TV by +84.5% and +106.8%), bone strength (maximum load by +30.5% and +31.6%) and bone histological properties (all *p* < 0.05 vs. PBS group). The therapeutic effects were similar among Apc001OA, Apc001OA‐d6, ALN and PTH 1–34 groups (all *p* > 0.05). After treatment with Apc001OA or Apc001OA‐d6, serum sclerostin levels significantly decreased by 25.0% and 24.9% (*p* < 0.05 vs. PBS group). The expression levels of key genes in the Wnt/β‐catenin pathway, Ctnnb1 and Lef1 significantly increased by 2.4‐ and 3.4‐fold in the Apc001OA group and by 2.5‐ and 3.5‐fold in the Apc001OA‐d6 group (*p* < 0.05 vs. PBS group), indicating that the aptamers improved bone properties through activating Wnt/β‐catenin pathway. Apc001OA and Apc001OA‐d6 significantly improved rotarod latency (*p* < 0.05 vs. PBS group) of ORX mice, and Apc001OA‐d6 could increase forelimb grip strength. Apc001OA, Apc001OA‐d6 and PTH 1–34 improved histological properties of muscle in ORX mice. No lesions or pathological changes were observed in the heart, aortic roots, liver, spleen, lungs or kidneys. Immunohistochemistry revealed no abnormal staining of interleukin 6 (IL‐6) and tumour necrosis factor‐α (TNF‐α) in the heart. There was no significant difference in serum concentrations of cardiac functional biomarkers, including creatine kinase‐MB (CK‐MB), cardiac troponin I (cTnI), B‐type natriuretic peptide (BNP) and inflammatory mediators (IL‐6 and TNF‐α) across all groups, indicating that Apc001OA and Apc001OA‐d6 had no adverse cardiovascular effects in ORX mice.

**Conclusions:**

The novel aptamers Apc001OA and Apc001OA‐d6, targeting sclerostin loop3, could significantly increase BMD and improve bone microarchitecture, bone biomechanics, muscle function and histological properties of muscle and bone in ORX mice, without adverse cardiovascular effects. These aptamers may serve as potential agents for treating osteoporosis and sarcopenia in men.

## 1 Introduction

Osteoporosis is a common skeletal disease characterized by low bone mineral density (BMD) and microarchitectural deterioration of bone tissue, leading to an increased risk of fragility fractures [1]. As individuals age, the prevalence of osteoporosis is increasing rapidly, bringing significant health and economic burdens. The number of male patients with osteoporotic fractures has markedly increased, and the remaining lifetime risk of hip fracture after 50 years old ranges from 6% to 14% in men [2]. Multiple studies demonstrate that the consequences of osteoporotic fractures in men are more severe than those in women, both in terms of morbidity and mortality [2, 3, 4]. However, osteoporosis remains underdiagnosed and undertreated in men, even after a fragility fracture [2]. Since therapeutic drugs for men with osteoporosis are limited, it is crucial to explore novel therapeutic drugs and molecules targeting the key regulatory pathways of male osteoporosis.

The Wnt/β‐catenin pathway and its bone‐specific inhibitor sclerostin play crucial roles in skeletal homeostasis [5]. A monoclonal antibody against sclerostin, romosozumab, can significantly increase the BMD of postmenopausal women and men with osteoporosis [6, 7]. However, romosozumab carries the potential risk of increased cardiovascular events, including cardiovascular death and myocardial infarction, which limits its long‐term and widespread use [8]. Therefore, it is essential to develop safe and effective new drugs targeting sclerostin for the treatment of male osteoporosis.

In addition, there is close crosstalk between bones and muscles, and osteoporosis is often accompanied by sarcopenia [9, 10]. Reduced muscle strength and muscle mass increase the risk of osteoporosis and fragility fractures [11, 12]. The Wnt pathway also exerts important roles in regulating the development and metabolism of muscle tissue [13, 14]. It is worthwhile to investigate whether drugs targeting the Wnt pathway also affect muscle tissue.

Recently, nucleic acid aptamers have been found to have pharmaceutical potential by selectively binding to specific target molecules [15]. A screened DNA aptamer (Apc001) and a 5‐quinoline modified aptamer have been shown to specifically promote bone anabolism, improve bone microarchitecture and enhance bone mechanical properties in ovariectomized (OVX) rats and osteogenesis imperfecta (OI) mice [16, 17, 18]. However, the effects of these aptamers on bone and muscle in men with osteoporosis remain unclear. Therefore, we investigated the effects of two aptamers targeting sclerostin loop3 (Apc001OA and Apc001OA‐d6) on the bones and muscles of orchiectomized (ORX) mice and explored their potential mechanisms. The effects of Apc001OA, Apc001OA‐d6, alendronate (ALN, a bone resorption inhibitor), teriparatide (PTH 1–34, a bone formation stimulator) and placebo (phosphate‐buffered saline, PBS) were further compared.

## 2 Methods

### 2.1 Animals, Treatment and Tissue Collection

A total of 48 male C57BL6 mice (10 weeks old, 22–26 g in body weight) were acclimated for 4 weeks in standard cages at an Association for Assessment and Accreditation of Laboratory Animal Care (AAALAC) accredited animal facility at Peking Union Medical College Hospital (PUMCH). All animal research procedures were approved by the Animal Welfare & Ethics Committee of PUMCH (Application No. XHDW‐2022‐068).

**FIGURE 1:**
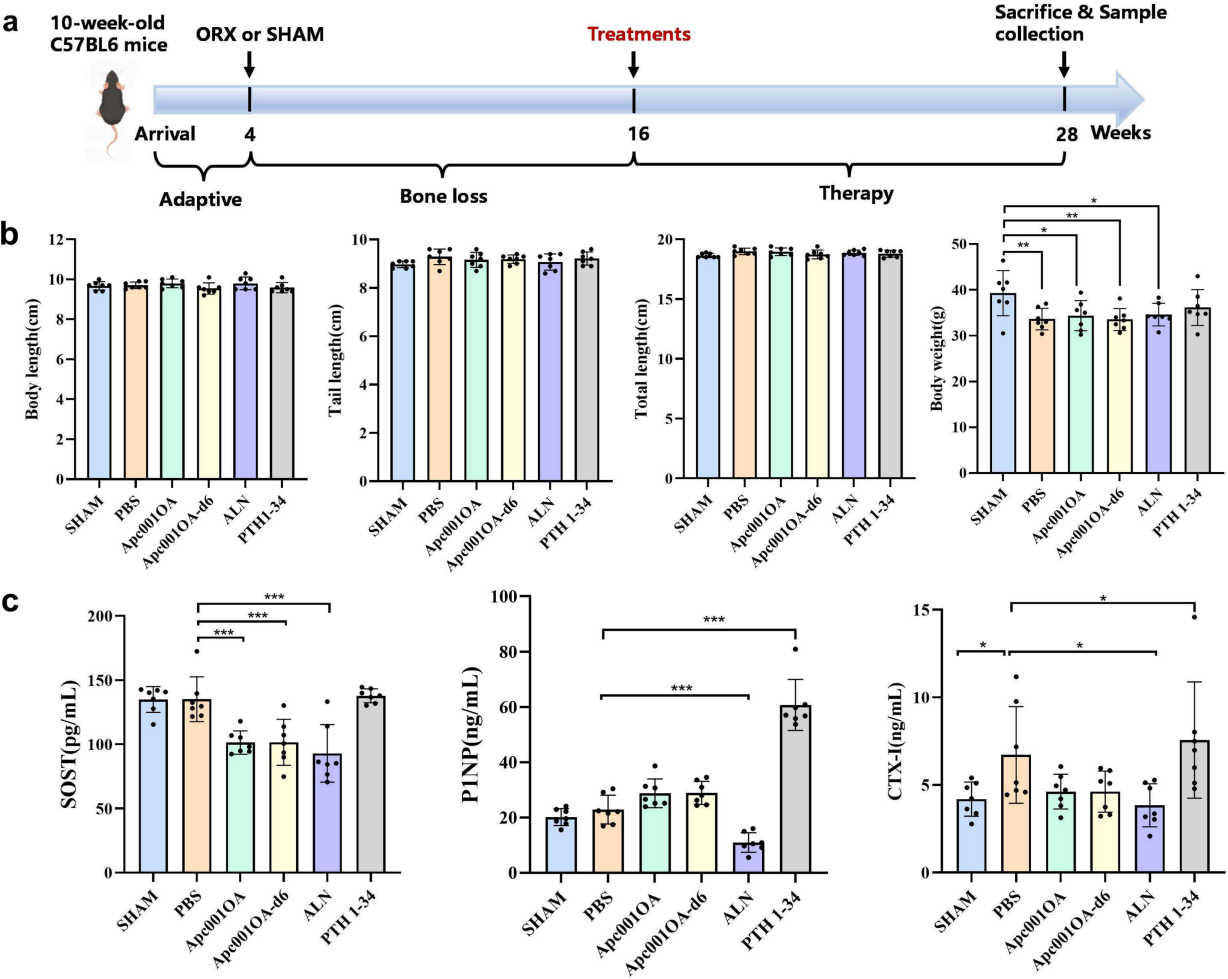
Schematic diagram of the animal study, characteristics of length, weight and bone turnover biomarkers of mice at euthanasia. (a) Schematic diagram of the animal study. (b) Characteristics of body length, tail length, total length and body weight at euthanasia. *: *p* < 0.05, **: *p* < 0.01, ***: *p* < 0.001 between the two groups. (c) Characteristics of bone turnover biomarkers at euthanasia. *: *p* < 0.05, **: *p* < 0.01, ***: *p* < 0.001 versus PBS group. ALN: alendronate, Apc001OA and Apc001OA‐d6 represent the two groups treated with the two sclerostin aptamers, CTX‐I: the C‐terminal cross‐linking telopeptide of Type I collagen, P1NP: the N‐terminal propeptide of Type 1 procollagen, PBS: phosphate‐buffered saline, PTH 1–34: teriparatide, SHAM: sham, SOST: sclerostin.

Bilateral ORX operation was performed on 40 mice, and sham (SHAM) surgery was performed on eight mice under general anaesthesia using 2% isoflurane at 14 weeks of age. After 12 weeks, the ORX mice were randomly divided into five groups to receive subcutaneous injections of the following agents (*n* = 8 per group): the aptamer groups (Apc001OA, 50 mg/kg, once a week, and Apc001OA‐d6, 50 mg/kg, once a week) [16, 17, 18], the positive control groups (ALN, 1 mg/kg, once a week, and PTH 1–34, 40 μg/kg/day, 5 days/week) [19] and the negative control group (PBS, 1 mL/kg, once a week). The doses and administration intervals for these drugs were determined based on previous studies [16, 17, 18, 19] and our prior experience with alendronate in mouse experiments. The aptamers of Apc001OA and Apc001OA‐d6 were obtained from the Law Sau Fai Institute for Advancing Translational Medicine in Bone and Joint Diseases (TMBJ), Hong Kong Baptist University [16, 17, 18, 20]. The structure diagram of the two aptamers Apc001OA and Apc001OA‐d6 is shown in Figure S1. PTH 1–34 was provided by Salubris Biotherapeutics Inc (Shenzhen, China), and ALN was provided by MSD Corporation (Merck and Co. Inc, Rahway, NJ, USA). After 12 weeks of treatment, all mice were euthanized. The mice were intraperitoneally injected with calcein (20 mg/kg, Sigma‐Aldrich, MO, USA) 3 and 10 days before euthanasia. Whole blood, internal organs (heart, liver, lung, spleen and kidneys) and bone and muscle tissues were collected (Figure 1a) after euthanasia.

### 2.2 Measurement of Serum Bone Turnover Biomarkers

The serum levels of bone turnover biomarkers were measured by enzyme‐linked immunosorbent assay (ELISA), including N‐terminal propeptide of Type 1 procollagen (P1NP, a bone formation marker, by Immunodiagnostic Systems Inc., Boldon, UK), C‐terminal cross‐linking telopeptide of Type I collagen (CTX‐I, a bone resorption marker, by Immunodiagnostic Systems Inc. Boldon, UK) and sclerostin (SOST, by R&D Systems Inc, USA). All procedures were performed according to the manufacturer's guidelines.

### 2.3 Measurement of BMD and Body Composition

BMD and body composition were measured by dual‐energy X‐ray absorptiometry (DXA, OsteoSys Co. Ltd) at 12 weeks after SHAM or ORX operation and at 4, 8 and 12 weeks of treatment with different drugs. Mice were anaesthetized, and full‐body scans were performed. Briefly, general BMD, bone mineral content (BMC), fat mass, lean mass, percentage of fat mass and lean mass and BMD at lumbar spine 1–5 (L1–5), femurs, tibia and fibula were calculated by extrapolating from rectangular region of interests (ROIs) drawn.

### 2.4 Analysis of Bone Microarchitecture

The bone microarchitecture of the left femur was analysed with microcomputed tomography (μCT45, Scanco Medical, Bassersdorf, Switzerland). Scans were performed with an X‐ray tube voltage of 70 kV, a current of 114 μA, an integration time of 300 ms and a voxel size of 10 μm. The ROI for trabecular bone was drawn manually using the Scanco evaluation software, starting from the growth plate of the distal epiphysis and extending 100 slices to the proximal end. Every measurement used the same filtering and segmentation values. Trabecular bone mineral density (Tb.vBMD), bone volume fraction (BV/TV), bone surface area per total volume (BS/TV), bone surface area per bone volume (BS/BV), trabecular number (Tb.N), trabecular thickness (Tb.Th), trabecular spacing (Tb.Sp), trabecular connectivity density (Tb.Conn) and trabecular structure model index (Tb.SMI) were calculated. Cortical bone was analysed in a 1000‐μm‐long volume situated in the middle of the diaphysis. Cortical bone mineral density (Ct.BMD) and cortical thickness (Ct.Th) were measured.

### 2.5 Biomechanical Property Testing

The right femurs, excised immediately after euthanasia, were frozen at −20°C in plastic bags and thawed at room temperature for the three‐point bending test on a fatigue‐testing machine (BOSE ElectroForce 3200, TA Instruments, New Castle, DE, USA) [21]. The three‐point bending tests were performed with the anterior surface of the femur resting on the bottom supports (span length: 8 mm) and the central load point moving at a rate of 1 mm/min until bone fractured. Force and displacement data were collected. The yield load, maximum load, breaking load and stiffness were calculated from the load–displacement curve.

### 2.6 Rotarod Test and Grip Strength Assessment

Motor coordination, strength and balance were assessed using a rotarod (Ugo Basile RotaRod, 47 650 Rota‐Rod NG, Ugo Basile, Italy) [22]. Forelimb and four limb grip strength were acquired with a grip strength meter (Ugo Basile 47200‐Grip‐Strength Meter, Ugo Basile, Italy) [22] at 0, 6 and 12 weeks of treatment.

Mice began training on the rotarod the day before data collection. They were placed onto the rod at a speed of 4 rpm, which accelerated over the course of 300 s to 40 rpm [23]. The latency to fall was recorded when the mouse fell from the rod. Each mouse was tested in three trials. The mean time from these trials was calculated for each mouse.

The mice were lowered onto a triangle bar or a grid grasping tool of the grip strength meter until they gripped the bar with their forelimbs or all four limbs. The mice were then gently pulled backward until they released their grip. The force gauge of the grip strength meter recorded the maximum force. Each animal was tested five times, and the mean of five values was recorded for each mouse.

### 2.7 Bone and Muscle Histomorphometric Analysis

Bone histological features were analysed by haematoxylin and eosin (H&E), Von Kossa's and tartrate‐resistant acid phosphatase (TRAP) staining. The left femur and the third and fourth vertebrae (L3–4) were decalcified after μCT scanning, embedded in paraffin and cut into 5‐μm sections using a microtome (Leica RM2016, Leica Microsystems), of which H&E and TRAP staining (Servicebio, Cat# G1050) were performed. The left tibia and the first and second vertebrae (L1–2) were fixed in 70% alcohol and embedded in modified methyl methacrylate without decalcification. The embedded samples were cut into 10‐μm‐thick sections, deplasticized and stained with a Von Kossa stain kit (Servicebio, Cat# G1043). For unstained slices, the bone mineral apposition rate (MAR) was calculated by dividing the distance between the two calcein labels by the interlabelling period. Analysis was performed with ImageJ software, following the recommendations of the ASBMR [24].

After euthanasia, soleus muscles were removed and fixed with 4% PFA for at least 24 h at 4°C. The muscles were dehydrated and then embedded in paraffin. Sections of 4.0 μm thick were obtained and stained with H&E. H&E sections were photographed under a microscope with a CCD camera. Cross‐sectional areas of at least 100 myofibers from the soleus muscles were quantified using ImageJ in a blinded manner.

### 2.8 Assessment of Cardiovascular Safety of the Treatment

After euthanasia, internal organs (including the heart, liver, spleen, lungs and kidneys) were removed and fixed with 4% PFA for at least 24 h at 4°C. The organs were dehydrated, embedded in paraffin, sectioned into 4.0‐μm‐thick slices and stained with H&E. Immunohistochemistry (IHC) staining for interleukin 6 (IL‐6) and tumour necrosis factor‐α (TNF‐α) in the heart was performed to assess the inflammatory status of cardiomyocytes.

Atherosclerotic plaque formation was quantified by measuring the surface area of Oil Red O‐positive lesions in en face preparations of the aortic roots. The saline‐perfused upper half of the heart, including the aortic root, was directly embedded in optimal cutting temperature (OCT) compound (Finetek Co. Ltd., Tokyo, Japan), frozen in liquid nitrogen and cryosectioned at 10‐μm thickness [17]. The ratio of atherosclerotic plaque area to total cross‐sectional area of aortic root was examined by Oil Red O staining.

Serum concentrations of cardiac functional biomarkers, including creatine kinase‐MB (CK‐MB), cardiac troponin I (cTnI) and B‐type natriuretic peptide (BNP), were quantified using commercial ELISA kits (Abcam and Thermo Fisher) under the manufacturer's standardized protocols. Serum levels of IL‐6 and TNF‐α were measured using ELISA kits (Thermo Fisher) following manufacturer's guidelines.

### 2.9 RNA Isolation and Real‐Time qPCR Analysis

Real‐time qPCR was used to evaluate the expression of related genes in the tibias. Right tibias were dissected free of soft tissues and ground into a powder in liquid nitrogen. Total RNA was extracted using TRIzol reagent, and the RNA was reverse transcribed into cDNA using the PrimeScript RT Reagent Kit (Takara, Kusatsu, Japan). The gene expression levels of Sost, Lrp5, Axin1, Ctnnb1, Lef1, Bglap, Runx2 and Sp7 were quantified by qPCR using TB Green Premix Ex Taq II (Tli RNase H Plus, Takara) on a Viia 7 Real‐Time PCR System (Life Technologies, USA). Primer sequences for RT‐qPCR are listed in Table S1. Relative messenger RNA expression levels were calculated using the 2^−ΔΔCT^ method and normalized to the internal control GAPDH.

### 2.10 Statistical Analysis

A paired‐sample Student's *t*‐test was used to longitudinally compare the differences in continuous variables between baseline and after treatment. Differences among the treatment groups were compared using one‐way ANOVA, followed by Tukey's post hoc test. Statistical analysis was performed using SPSS Statistics 26.0 (IBM, Armonk, NY, USA), GraphPad Prism 8 (Statcon). Statistical significance was defined as a *p*‐value of ≤ 0.05.

## 3 Results

### 3.1 The Model of Male Osteoporosis With Sarcopenia Was Generated

After 12 weeks of ORX or SHAM operation, general BMD, lumbar, femoral and tibial BMD were significantly lower in ORX mice compared with SHAM mice (Table 1), confirming the successful generation of the male osteoporosis model. Additionally, lean mass and percentage of lean mass were significantly lower in ORX mice than in SHAM mice, whereas fat mass and percentage of fat mass were higher in ORX mice than in SHAM mice. The rotarod test showed that the time to fall in ORX mice was significantly shorter than in SHAM mice, and grip strength, both for the forelimbs and the four limbs, was lower in ORX mice compared with SHAM mice (Table 1). These results indicate that the male osteoporosis model with sarcopenia was successfully generated by ORX.

**TABLE 1:** Characteristics of BMD, body composition, rotarod test and grip strength after 12 weeks of SHAM and ORX operation.

	ORX (*n* = 35)	SHAM (*n* = 7)	*p*
General BMD (mg/cm^2^)	72.8 ± 4.4	82.0 ± 1.6	**< 0.001**
BMC (g)	0.617 ± 0.050	0.623 ± 0.098	0.826
BMD at lumbar spine (mg/cm^2^)	43.9 ± 3.3	50.7 ± 4.2	**< 0.001**
BMD at left femur (mg/cm^2^)	77.5 ± 5.1	86.1 ± 6.0	**< 0.001**
BMD at right femur (mg/cm^2^)	78.7 ± 4.4	87.3 ± 7.9	**< 0.001**
BMD at left tibia and fibula (mg/cm^2^)	45.2 ± 4.2	55.1 ± 6.3	**< 0.001**
BMD at right tibia and fibula (mg/cm^2^)	45.1 ± 5.8	56.7 ± 1.7	**< 0.001**
Body composition			
Fat (g)	8.29 ± 1.40	6.87 ± 1.83	**< 0.001**
Fat (%)	24.55 ± 2.31	19.95 ± 3.59	**0.024**
Lean (g)	25.26 ± 1.52	27.14 ± 2.20	**0.008**
Lean (%)	74.08 ± 2.23	78.62 ± 3.57	**< 0.001**
Time to fall (s) in Rotarod test	222.4 ± 27.0	262.1 ± 20.6	**0.001**
Four limbs grip strength (g)	140.9 ± 10.7	166.6 ± 14.2	**< 0.001**
Forelimbs grip strength (g)	49.6 ± 6.8	63.9 ± 9.1	**< 0.001**

*Note:* Bold values indicate that there was a signification difference between the two groups.

Abbreviations: BMC: bone mineral content; BMD: bone mineral density; ORX: orchiectomy; SHAM: sham.

### 3.2 Effect of Sclerostin Aptamers on Bone in ORX Mice

Figure 1a shows the schematic diagram of this study. After 12 weeks of treatment, body length, tail length and total body length were similar across all treatment groups and the SHAM group. Total body weight was significantly higher in the SHAM group than placebo, Apc001OA, Apc001OA‐d6 and ALN groups, with no significant difference observed between SHAM and PTH1–34 groups (Figure 1b).

#### 3.2.1 Changes in Bone Turnover Biomarkers

After 12 weeks of treatment, serum sclerostin levels were significantly decreased by 25.0%, 24.9% and 31.3% in the Apc001OA, Apc001OA‐d6 and ALN groups compared with the PBS group (all *p* < 0.001), with no significant difference between the PTH 1–34 and PBS groups (Figure 1c). Serum levels of P1NP and CTX‐I were significantly reduced in the ALN group, whereas serum P1NP and CTX‐I levels were significantly increased in the PTH 1–34 group. Compared with the PBS group, serum P1NP levels showed an increasing trend in the Apc001OA and Apc001OA‐d6 groups, and CTX‐I levels appeared to decrease in these groups, but neither change reached statistical significance (Figure 1c).

#### 3.2.2 Changes in Bone Microarchitecture

**FIGURE 2:**
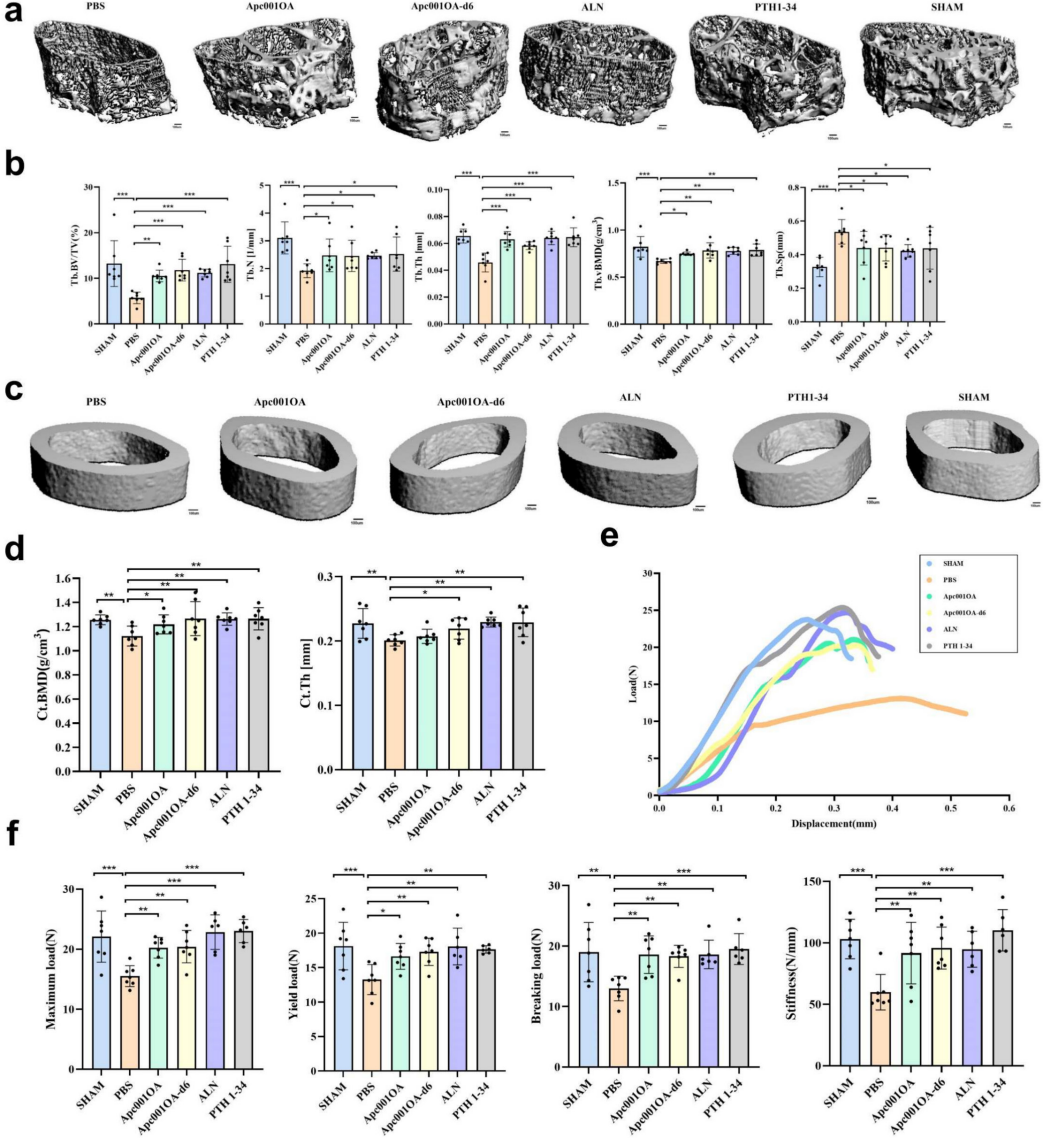
Characteristics of bone microarchitecture properties and bone biomechanical properties of mice at euthanasia. (a) Typical images for trabecular bone microarchitecture of mice after 12 weeks of treatment, Scale bar = 100 μm. (b) Characteristics of trabecular bone microarchitecture properties of mice after 12 weeks of treatment. (c) Typical images for cortical bone microarchitecture of mice after 12 weeks of treatment, Scale bar = 100 μm. (d) Characteristics of cortical bone microarchitecture properties of mice after 12 weeks of treatment. (e) Typical load–displacement curves of mice after 12 weeks of treatment. (f) Characteristics of bone biomechanical properties of mice after 12 weeks of treatment. *: *p* < 0.05, *: *p* < 0.01, ***: *p* < 0.001 versus PBS group. ALN: alendronate, Apc001OA and Apc001OA‐d6 represent the two groups treated with the two sclerostin aptamers, Ct.BMD: cortical bone mineral density, Ct.Th: cortical thickness, PBS: phosphate‐buffered saline, PTH 1–34: teriparatide, SHAM: sham, Tb.BV/TV: trabecular bone volume per total volume, Tb.N: trabecular number, Tb.Sp: trabecular separation, Tb.Th: trabecular thickness, Tb.vBMD: trabecular bone mineral density.

After 12 weeks of treatment, the Apc001OA group showed significantly higher BV/TV (+84.5%, *p* < 0.01), BS/TV (+28.6%, *p* < 0.05), Tb.N (+28.9%, *p* < 0.05), Tb.Th (+37.8%, *p* < 0.001), Tb.vBMD (+11.9%, *p* < 0.05) and Tb.Conn.D (+37.4%, *p* < 0.05) and lower BS/BV (−32.3%, *p* < 0.001) and Tb.Sp (−18.2%, *p* < 0.05) compared with the PBS group (Figures 2a–b and S2). Treatment with Apc001OA‐d6 significantly increased BV/TV (+106.8%, *p* < 0.001), BS/TV (+44.5%, *p* < 0.001), Tb.N (+27.9%, *p* < 0.05), Tb.Th (+27.4%, *p* < 0.001), Tb.vBMD (+17.1%, *p* < 0.01) and Tb.Conn.D (+40.5%, *p* < 0.01) and reduced BS/BV (−31.9%, *p* < 0.001) and Tb.Sp (−17.8%, *p* < 0.05) compared with the PBS group. No significant differences were found between the Apc001OA and Apc001OA‐d6 groups for the above parameters. The ALN and PTH 1–34 treatments also significantly increased BV/TV, BS/TV, Tb.N, Tb.Th, Tb.vBMD and Tb.Conn.D, while decreasing BS/BV and Tb.Sp compared with the PBS group (Figures 2a–b and S2). The effects of the two sclerostin aptamers on bone microarchitecture were similar to those of ALN and PTH 1–34. The average absolute value of Tb.SMI was closer to 0 in the Apc001OA‐d6 and PTH 1–34 groups, whereas it was closer to 3 in the PBS group (both *p* < 0.05), indicating a shift from rod‐like to plate‐like trabecular bone structure in the left femur of ORX mice after 12 weeks of Apc001OA‐d6 and PTH 1–34 treatment (Figure S2).

For cortical bone, treatment with Apc001OA and Apc001OA‐d6 for 12 weeks significantly increased Ct.BMD (all *p* < 0.05 vs. PBS group). Apc001OA‐d6 also significantly improved Ct.Th compared with the PBS group, whereas Apc001OA had no significant effect on Ct.Th (Figure 2c–d). Both ALN and PTH 1–34 treatments significantly improved Ct.Th and Ct.BMD, with values higher than those in the PBS group. There were no significant differences in Ct.Th and Ct.BMD among the Apc001OA‐d6, ALN and PTH 1–34 groups.

#### 3.2.3 Changes in Bone Biomechanical Parameters

In comparison with the PBS group, the load–displacement curve (Figure 2e) of the femoral three‐point mechanical test indicated that the maximum load (+30.5% and +31.6%, *p* < 0.01), yield load (+25.5% and +30.5%, *p* < 0.01), breaking load (+43.0% and +40.9%, *p* < 0.001) and stiffness (+53.2% and +60.1%, *p* < 0.001) were significantly increased after 12 weeks of Apc001OA and Apc001OA‐d6 treatment, with no significant differences between the Apc001OA and Apc001OA‐d6 groups. ALN and PTH 1–34 treatment also significantly improved bone strength and stiffness, and no significant differences in maximum load, yield load, breaking load and stiffness were found among the Apc001OA, Apc001OA‐d6, ALN, PTH 1–34 and SHAM groups (Figure 2f).

#### 3.2.4 Changes in Bone Histological Properties

**FIGURE 3:**
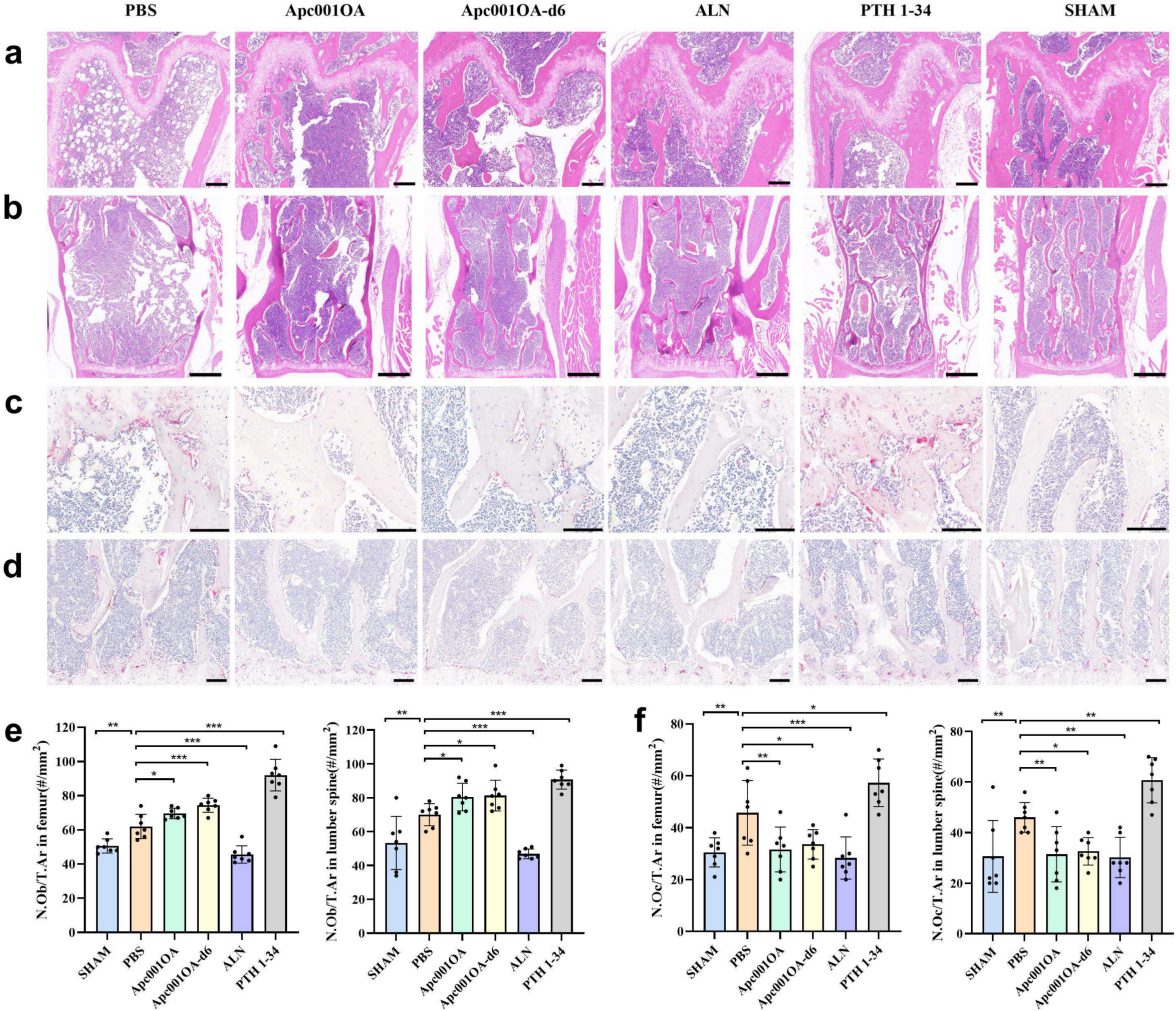
Characteristics of bone histological properties of mice after 12 weeks of treatment. (a) H&E staining of the left femur of mice after 12 weeks of treatment, Scale bar = 200 μm. (b) H&E staining of the L3–4 of mice after 12 weeks of treatment, Scale bar = 500 μm. (c)TRAP staining of the left femur of mice after 12 weeks of treatment, Scale bar = 100 μm. (d) TRAP staining of the L3–4 of mice after 12 weeks of treatment, Scale bar = 100 μm. (e) Quantitative analysis of osteoblast number relative to tissue area (N.Ob/T.Ar, #/mm^2^) in femur and lumber spine after 12 weeks of treatment. (f) Quantitative analysis of number of TRAP‐positive osteoclasts related to tissue area (N.Oc/T.Ar, #/mm^2^) in femur and lumber spine after 12 weeks of treatment. *: *p* < 0.05, **: *p* < 0.01, ***: *p* < 0.001 versus PBS group. ALN: alendronate, Apc001OA and Apc001OA‐d6 represent the two groups treated with the two sclerostin aptamers, H&E: haematoxylin and eosin, N.Ob/T.Ar: osteoblast number relative to tissue area, N.Oc/T.Ar: number of TRAP‐positive osteoclasts related to tissue area, PBS: phosphate‐buffered saline, PTH 1–34: teriparatide, SHAM: sham, TRAP: tartrate resistant acid phosphatase.

In H&E staining images, the PBS group showed sparse loss of trabecular interconnectivity and thinning of the trabeculae at the femur and lumbar spine, resulting in widened intertrabecular spaces over time. Treatment with Apc001OA, Apc001OA‐d6, ALN and PTH 1–34 increased trabecular area and thickness and decreased trabecular separation (Figure 3a–b). The number of osteoblasts was significantly increased in the Apc001OA, Apc001OA‐d6 and PTH 1–34 groups but significantly decreased in the ALN group (Figure 3e). In the femur and lumbar vertebrae (L3–4), TRAP activity showed that the number of osteoclasts was significantly higher in ORX mice than in the SHAM group (Figure 3c–d). The number of osteoclasts was significantly increased in the PTH 1–34 group and significantly decreased in the Apc001OA, Apc001OA‐d6 and ALN groups (Figure 3f).

**FIGURE 4:**
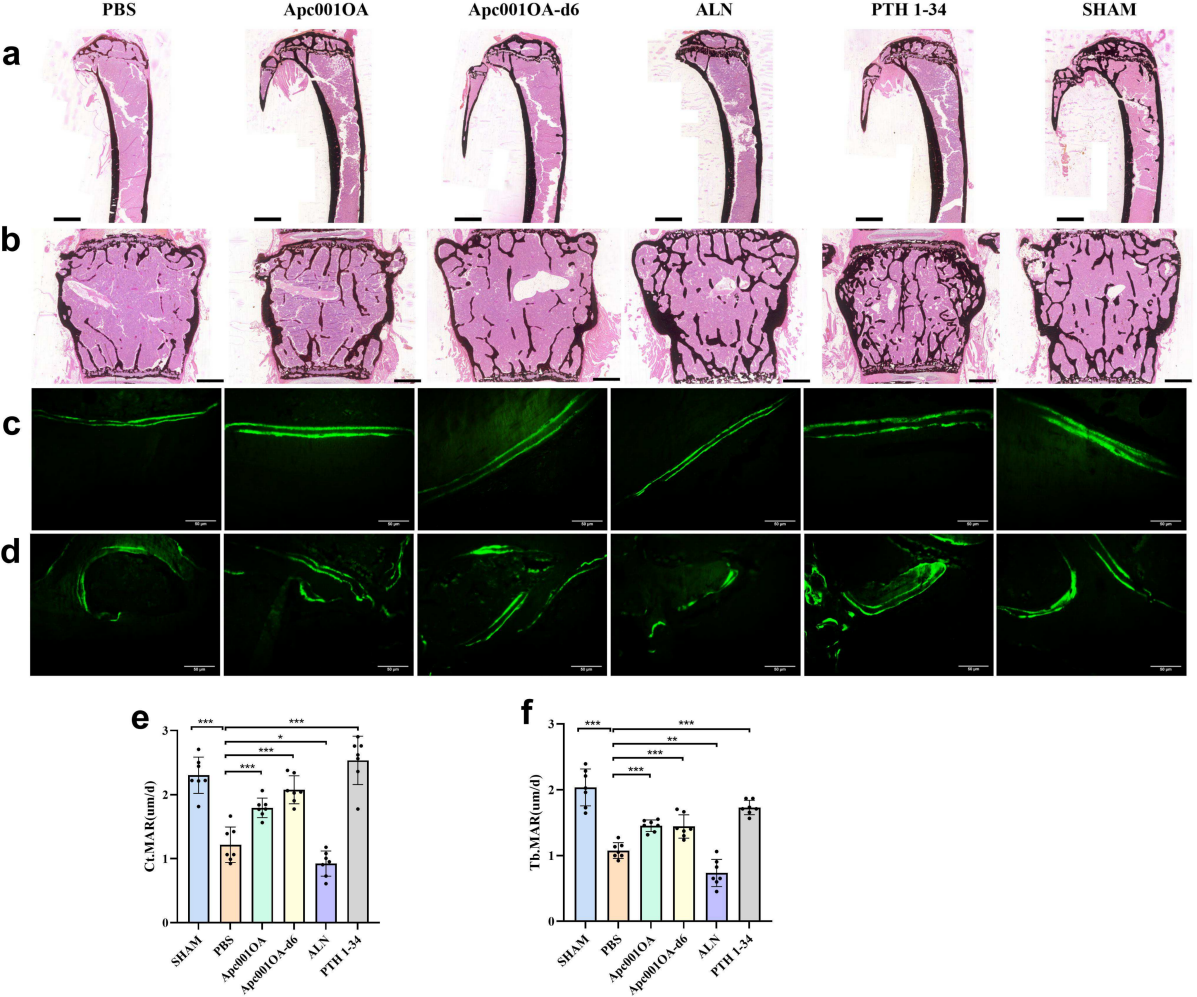
Characteristics of bone histological properties and bone formation rate of mice after 12 weeks of treatment. (a) Von Kossa's staining of the left tibia of mice after 12 weeks of treatment, Scale bar = 1000 μm. (b) Von Kossa's staining of the L1–2 of mice after 12 weeks of treatment, Scale bar = 500 μm. (c) Typical images of unstained and uncalcified tibia showing Ct.MAR of mice after 12 weeks of treatment, Scale bar = 50 μm. (d) Typical images of unstained and uncalcified vertebra of mice showing Tb.MAR after 12 weeks of treatment, Scale bar = 50 μm. (e) Comparison of Ct.MAR of the left tibia of mice after 12 weeks of treatment. (f) Comparison of Tb.MAR of the vertebra of mice after 12 weeks of treatment. *: *p* < 0.05, **: *p* < 0.01, ***: *p* < 0.001 versus PBS group. ALN: alendronate, Apc001OA and Apc001OA‐d6 represent the two groups treated with the two sclerostin aptamers, Ct.MAR: cortical mineral apposition rate, PBS: phosphate‐buffered saline, PTH 1–34: teriparatide, SHAM: sham, Tb.MAR: trabecular mineral apposition rate.

In Von Kossa's staining images, reduced trabecular area and trabecular thickness were observed in tibia and lumbar spine in ORX group (Figure 4a–b). Treatment with Apc001OA, Apc001OA‐d6, ALN and PTH 1–34 significantly increased both trabecular area and trabecular thickness. In bone histomorphometric analysis of tibial cortical bone (Figure 4c) and lumbar trabecular bone (Figure 4d), cortical mineral apposition rate (Ct.MAR) in the tibia was notably higher in the Apc001OA, Apc001OA‐d6 and PTH 1–34 groups compared with the PBS group (Figure 4e). The lumbar trabecular MAR (Tb.MAR) was significantly increased in the Apc001OA, Apc001OA‐d6 and PTH 1–34 groups but significantly decreased in the ALN group compared with the PBS group (Figure 4f).

#### 3.2.5 Changes in the Expression of Target Genes of the Wnt Pathway

**FIGURE 5:**
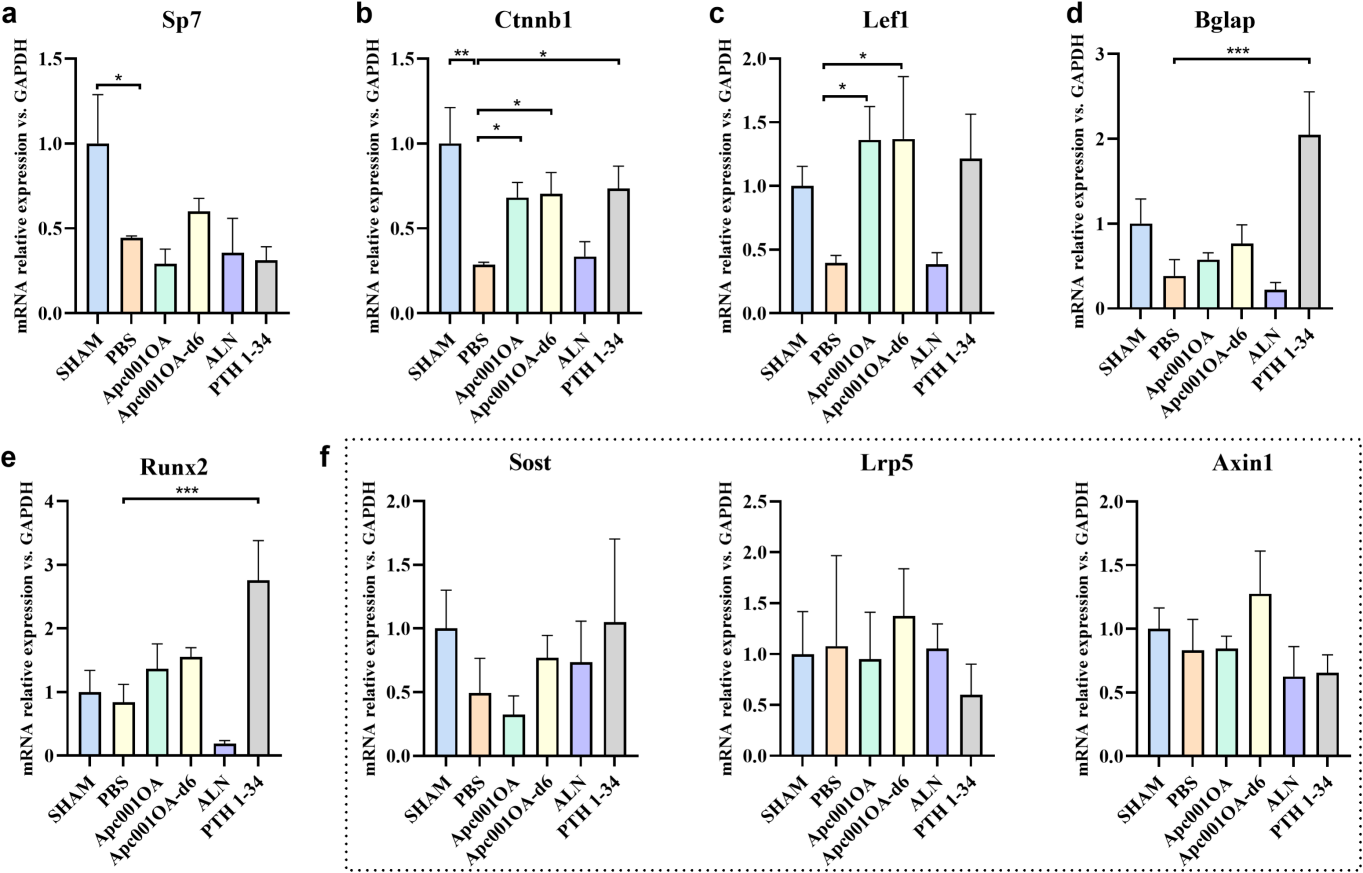
Relative gene expression in the tibias from mice after 12 weeks of treatment. (a) Relative gene expression of Sp7 versus Gapdh of mice after 12 weeks of treatment. (b) Relative gene expression of Ctnnb1 versus Gapdh of mice after 12 weeks of treatment. (c) Relative gene expression of Lef1 versus Gapdh of mice after 12 weeks of treatment. (d) Relative gene expression of Bglap versus Gapdh of mice after 12 weeks of treatment. (e) Relative gene expression of Runx2 versus Gapdh of mice after 12 weeks of treatment. (f) Relative gene expression of Sost, Lrp5 and Axin1 versus Gapdh of mice after 12 weeks of treatment. *: *p* < 0.05, **: *p* < 0.01, ***: *p* < 0.001 versus PBS group. ALN: alendronate, Apc001OA and Apc001OA‐d6 represent the two groups treated with the two sclerostin aptamers, PBS: phosphate‐buffered saline, PTH 1–34: teriparatide, SHAM: sham.

The expression levels of Sp7 and Ctnnb1 were significantly decreased in the PBS group compared with the SHAM group (Figure 5a–b), indicating that the Wnt/β‐catenin pathway was impaired. After 12 weeks of treatment, the expression levels of Ctnnb1 and Lef1 were significantly increased by 2.4‐ and 3.4‐fold in the Apc001OA group and 2.5‐ and 3.5‐fold in the Apc001OA‐d6 group compared with the PBS group (*p* < 0.05), suggesting that aptamers could improve skeletal properties by activating the Wnt/β‐catenin pathway (Figure 5b–c). The expression levels of Bglap and Runx2 in the PTH1–34 group were significantly higher than in the PBS group, indicating that PTH1–34 treatment could promote bone formation (Figure 5d–e). The expression levels of Bglap and Runx2 in the Apc001OA and Apc001OA‐d6 groups showed an increasing trend compared with the PBS group, although they did not reach statistical significance (Figure 5d–e). The expression levels of Sost, Lrp5 and Axin1 were similar across all groups (Figure 5f).

### 3.3 Effects of Apc001OA and Apc001OA‐d6 on Muscle Function in Mice

**FIGURE 6:**
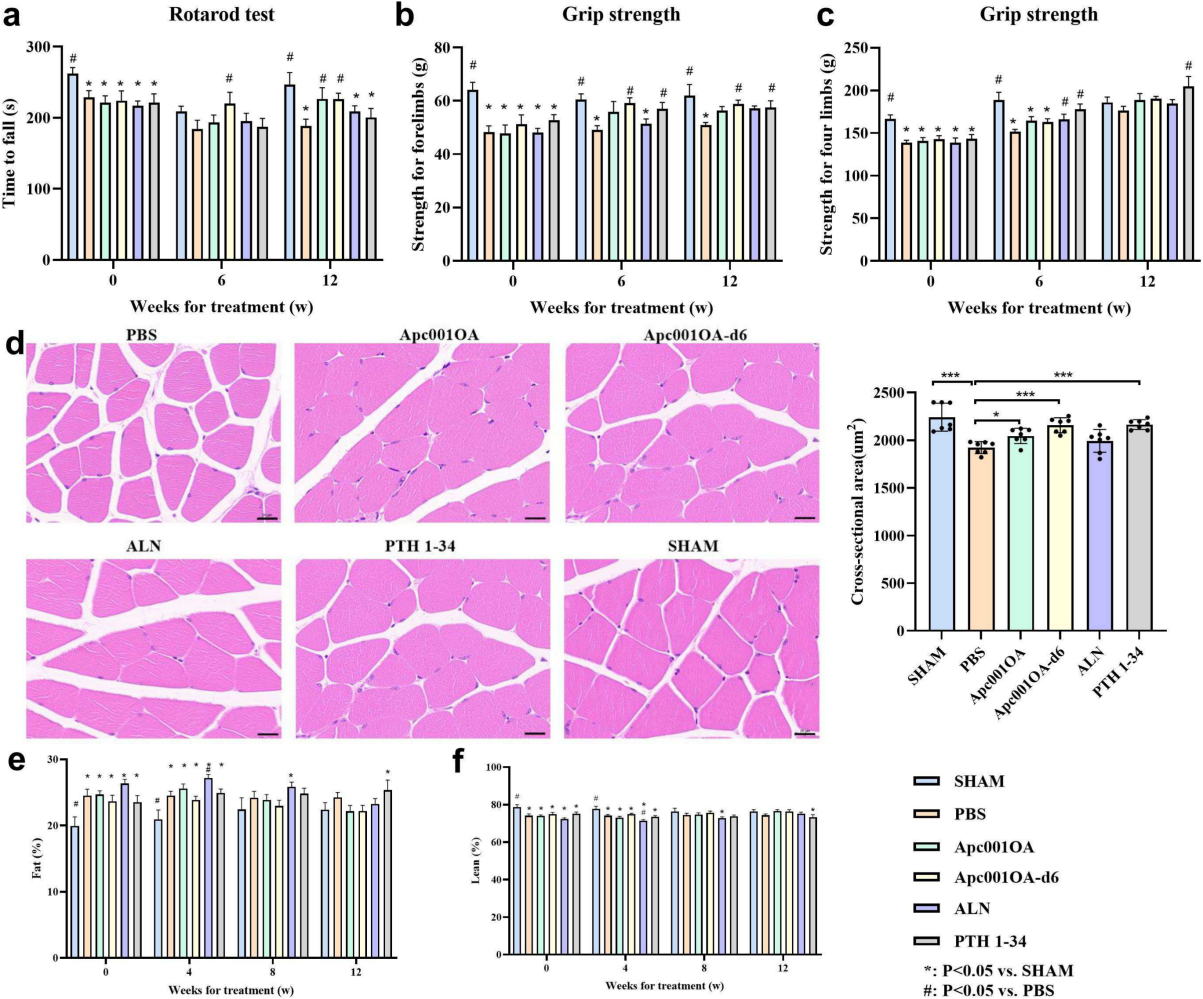
Muscular characteristics of the mice during the treatment period. (a) Rotarod test of the mice after 0, 6 and 12 weeks of treatment. *: *p* < 0.05 versus SHAM group, #: *p* < 0.05 versus PBS group. (b) Grip strength for forelimbs of the mice after 0, 6 and 12 weeks of treatment. *: *p* < 0.05 versus SHAM group, #: *p* < 0.05 versus PBS group. (c) Grip strength for four limbs of the mice after 0, 6 and 12 weeks of treatment. *: *p* < 0.05 versus SHAM group, #: *p* < 0.05 versus PBS group. (d) Typical images of H&E staining and cross‐sectional area of the soleus muscle of mice after 12 weeks of treatment, Scale bar = 20 μm, *: *p* < 0.05, **: *p* < 0.01, ***: *p* < 0.001 versus PBS group. (e) The percent of fat mass of the mice after 0, 6, 8 and 12 weeks of treatment. *: *p* < 0.05 versus SHAM group, #: *p* < 0.05 versus PBS group. (f) The percent of lean mass of the mice after 0, 6, 8 and 12 weeks of treatment. *: *p* < 0.05 versus SHAM group, #: *p* < 0.05 versus PBS group. ALN: alendronate, Apc001OA and Apc001OA‐d6 represent the two groups treated with the two sclerostin aptamers, H&E: haematoxylin and eosin, PBS: phosphate‐buffered saline, PTH 1–34: teriparatide, SHAM: sham.

After 12 weeks of treatment, the Apc001OA and Apc001OA‐d6 groups had a longer time to fall in the rotarod test compared with the PBS group, indicating that aptamer treatment may improve motor coordination and balance in mice (Figure 6a). Interestingly, the grip strength of the forelimbs in the Apc001OA‐d6 group was significantly higher than in the PBS group (Figure 6b), but there was no significant difference between the Apc001OA group and the PBS group (Figure 6b). There was no significant difference in the grip strength of all four limbs among the Apc001OA, Apc001OA‐d6 and PBS groups (Figure 6c). PTH 1–34 treatment also improved the grip strength of all four limbs and forelimbs compared with the PBS group (Figure 6b–c).

The histological sections showed that the cross‐sectional area of the soleus muscle was significantly lower in ORX mice compared with SHAM mice at execution. After 12 weeks of treatment with Apc001OA, Apc001OA‐d6 and PTH 1–34, the cross‐sectional areas of the soleus muscle were significantly higher than in the PBS group (Figure 6d). No significant differences were found in body composition by DXA among the Apc001OA, Apc001OA‐d6 and PBS groups (Figure 6e–f).

### 3.4 Cardiovascular Effects of Apc001OA and Apc001OA‐d6 on Mice

**FIGURE 7:**
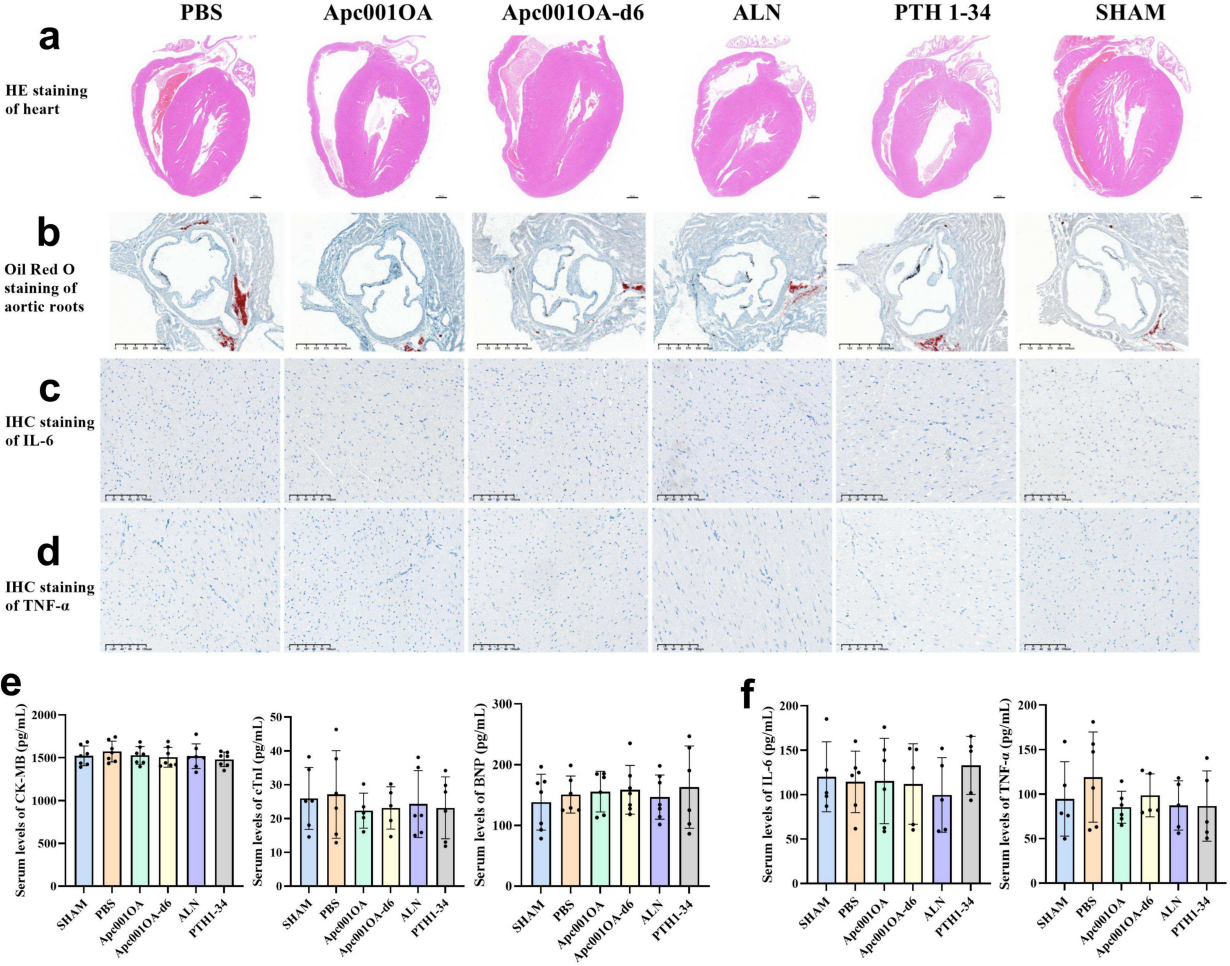
Assessment of cardiovascular safety of the treatment. (a) H&E staining of the heart of mice after 12 weeks of treatment, Scale bar = 500 μm. (b) Oil Red O staining of the aortic roots of heart of mice after 12 weeks of treatment, Scale bar = 625 μm. (c) IHC staining of IL‐6 on heart sections of mice after 12 weeks of treatment, Scale bar = 100 μm. (d) IHC staining of TNF‐α on heart sections of mice after 12 weeks of treatment, Scale bar = 100 μm. (e) Serum concentrations of cardiac functional biomarkers of mice after 12 weeks of treatment. (f) Serum levels of inflammatory cytokines of mice after 12 weeks of treatment. ALN: alendronate, Apc001OA and Apc001OA‐d6 represent the two groups treated with the two sclerostin aptamers, BNP: B‐type natriuretic peptide, CK‐MB: creatine kinase‐MB, cTnI: cardiac troponin I, H&E: haematoxylin and eosin, IHC: immunohistochemistry, IL‐6: interleukin 6, PBS: phosphate‐buffered saline, PTH 1–34: teriparatide, SHAM: sham, TNF‐α: tumour necrosis factor‐α.

After 12 weeks of treatment, normal histological shape and structure of the heart were observed in all groups in H&E staining (Figure 7a). The cardiomyocytes were normally branched and interconnected, exhibiting striations with a typical, orderly arrangement of myofibrils. Microscopic examination revealed the normal structure of the cardiomyocytes, with no lesions or pathological changes (Figure 7a). Oil Red O staining showed no lesions on the aortic roots of any mice (Figure 7b), indicating that Apc001OA and Apc001OA‐d6 treatments did not increase the risk of atherosclerosis in ORX mice. IHC staining of IL‐6 and TNF‐α in the heart also showed no obvious abnormalities (Figure 7c–d), suggesting that there was no excessive inflammatory response in the cardiomyocytes of any mice. There was no significant difference in serum concentrations of cardiac functional biomarkers among the six groups, including CK‐MB, cTnI and BNP (Figure 7e). There were no significant differences in serum levels of inflammatory cytokines among the groups, including IL‐6 and TNF‐α (Figure 7f). These results may suggest that Apc001OA and Apc001OA‐d6 treatments had no adverse cardiovascular effects in ORX mice.

Additionally, the liver, spleen, lungs and kidneys showed normal cell structure in H&E staining in all groups after 12 weeks of treatment (Figure S3a–d). These results suggest that Apc001OA and Apc001OA‐d6 treatments were safe for all important internal organs.

## 4 Discussion

We evaluated the effects of two novel aptamers targeting sclerostin, Apc001OA and Apc001OA‐d6, on the bones and muscles of ORX mice for the first time. The results showed that treatment with Apc001OA and Apc001OA‐d6 (50 mg/kg/week) significantly increased BMD, improved the microarchitecture of trabecular and cortical bone and promoted skeletal biomechanical and histological properties, similar to those of ALN and PTH 1–34. Notably, Apc001OA and Apc001OA‐d6 treatment improved motor coordination, balance and muscular histological properties in ORX mice, with Apc001OA‐d6 also increasing the grip strength of the forelimbs. We further confirmed that Apc001OA and Apc001OA‐d6 improved bone and muscle parameters by activating the Wnt/β‐catenin pathway and elevating the expression of key genes in this pathway. Importantly, Apc001OA and Apc001OA‐d6 had no adverse effects on the cardiovascular system of ORX mice.

Sclerostin is a glycoprotein secreted by osteocytes, partially in response to mechanical loading, and consists of three loops: loop1, loop2 and loop3 [25]. Sclerostin directly inhibits bone formation by blocking the Wnt pathway and indirectly accelerates bone resorption by osteoclasts through reduced osteoprotegerin secretion in osteoblasts [26]. The therapeutic potential of antisclerostin antibodies, romosozumab, has a dual effect of promoting bone formation and inhibiting bone resorption. It has been used to increase BMD and reduce the incidence of fragility fractures in postmenopausal women and individuals with male osteoporosis [6, 7, 27, 28, 29]. However, romosozumab has been associated with a potential risk of increasing cardiac ischemic events [6, 27, 28], which limits its long‐term and widespread use.

Sclerostin can interact with different transmembrane receptors via its various loops to play distinct roles in inhibiting bone formation and protecting the cardiovascular system [16, 17]. Sclerostin loop3 is involved in inhibiting bone formation but does not affect the cardiovascular system [16]. Aptamers are synthetic, single‐stranded DNA or RNA molecules isolated from combinatorial oligonucleotide libraries, and they have affinities and specificities similar to monoclonal antibodies [30, 31]. The newly identified aptamer, aptscl56, showed a high binding ability to sclerostin loop3 but not loop1 and loop2 [16, 17]. However, its druggability was limited by a very short half‐life because of its molecular weight being below the cutoff threshold for renal filtration. Modified aptscl56 conjugated to polyethylene glycol (PEG40k‐aptscl56, named Apc001PE) or octadecanedioic acid (OA, Apc001OA) can protect against renal filtration, resulting in a longer half‐life [16, 18]. The PEG moiety constitutes a substantial portion of the PEG‐aptamer conjugate (over 75%). This proportion poses challenges in augmenting the subcutaneous dosage of the aptamer given a fixed administration volume, significantly restricting its therapeutic potential. OA, a fatty acid (FA), has a strong binding affinity for human serum albumin (HSA) and a molecular mass that makes it suitable for modification. The modification strategy of OA–aptamer conjugate can significantly increase the proportion of aptamer within the aptamer conjugate (up to 90%) than PEGylation (low to 25%), thus increasing the subcutaneous dosage for the aptamer moiety at a fixed subcutaneous administration volume. Previous studies demonstrated that sclerostin aptamer Apc001PE could promote bone formation in the OVX‐induced osteoporotic rats and OI mice [16, 17]. Apc001OA has been shown to promote bone formation, increase bone mass and improve bone microarchitecture integrity without causing cardiac adverse events in OI mice [18, 20]. Apc001OA‐d6 is a sclerostin aptamer with a quinoline modification at the 6th site of Apc001OA to enhance its binding ability. The effects of sclerostin aptamers on ORX animal models have not yet been reported.

Our results showed that Apc001OA and Apc001OA‐d6 significantly increased BMD, improved trabecular microarchitecture, promoted biomechanics and enhanced histological properties of bone in ORX mice. These effects were similar to those of ALN and PTH1–34 in ORX mice, as well as to the effects of Apc001PE on bone in OVX rats [16, 17]. The serum levels of P1NP showed an increasing trend, whereas the serum levels of CTX‐I appeared to decrease in the Apc001OA and Apc001OA‐d6 groups compared with the PBS group. Serum sclerostin levels were significantly lower in the Apc001OA and Apc001OA‐d6 groups than in the PBS group, indicating that Apc001OA and Apc001OA‐d6 are dual‐action agents. Sclerostin can bind to low‐density lipoprotein receptor–related protein 5/6 (LRP5/6) and inhibit bone anabolic Wnt signalling [32]. In the absence of the Wnt inhibitor sclerostin, Wnt proteins bind to and activate the heterodimeric receptor complex FZD‐LRP5/6, resulting in the dissociation of the ‘destruction complex’ from β‐catenin. This leads to the subsequent accumulation of unphosphorylated β‐catenin in the cytoplasm, followed by translocation of this active form of β‐catenin into the nucleus. Intranuclear β‐catenin associates with members of the T cell factor/lymphoid enhancer factor (TCF/LEF) family, thus regulating osteogenic differentiation and bone formation [5]. qPCR analysis showed that the expression levels of Ctnnb1 and Lef1 in the Apc001OA and Apc001OA‐d6 groups were significantly higher, indicating that Apc001OA and Apc001OA‐d6 could bind to sclerostin protein, activate the Wnt/β‐catenin pathway and increase downstream gene expression, thereby promoting bone anabolism.

We found no significant differences in the effects of Apc001OA and Apc001OA‐d6 on trabecular bone microarchitecture, bone biomechanics and bone histological properties. However, a previous study showed that the Apc001OA‐d6 25 mg/kg/week (d6OA‐25) treatment group had significantly higher Tb.Conn.D, Tb.vBMD and Tb. N and failure force in compression tests compared with the Apc001OA 25 mg/kg/week (OA‐25) group in OI mice [20]. They demonstrated that over 17‐fold enhancement of binding affinity by 5‐quinoline modification in Apc001OA‐d6 and 5‐quinoline modification could facilitate the modified aptamer attenuating the suppressed effect of the transfected sclerostin on both Wnt signalling in vitro, which was almost the same level of romosozumab in OI mice [20]. The differences in our results may be attributed to the use of different animal models of male osteoporosis, different ages of mice and different intervention doses of aptamers. For cortical bone, treatment with Apc001OA‐d6 improved Ct.BMD and Ct.Th, whereas Apc001OA only promoted Ct.BMD compared with PBS. This may suggest that Apc001OA‐d6 is more effective than Apc001OA in promoting cortical bone formation. Therefore, the 5‐quinoline modification could help address the druggability bottleneck of nucleic acid aptamers with low affinity.

Osteoporosis often co‐occurs with sarcopenia because of the close crosstalk between muscles and bones [33, 34, 35]. The significant results of this study show that Apc001OA and Apc001OA‐d6 can improve motor coordination, balance and histological properties of muscle in ORX mice. Apc001OA‐d6 treatment also increased the grip strength of forelimbs. A previous study indicated that romosozumab may reduce the risk of falls in postmenopausal women with osteoporosis [36]. WNT/β‐catenin signalling also plays a critical role in the development of muscle tissue [37]. In myogenesis, the effect of Wnt signalling leads to the progression of the differentiation at early developmental stages and inhibition of this signalling results in a poor skeletal muscle formation [37]. In muscle fibrosis, Wnt signalling can functionally interact with other profibrotic molecules [37]. The possible mechanism through which Apc001OA and Apc001OA‐d6 affect muscle is that they can activate the WNT/β‐catenin signalling pathway, regulate myogenesis and muscle fibrosis and subsequently improve muscle function and strength. The exact mechanism, however, warrants further exploration. Moreover, histological examination revealed a normal cellular structure and no lesions in the internal organs following treatment with Apc001OA and Apc001OA‐d6, suggesting that the two sclerostin aptamers are safe for the internal organs of male osteoporosis mice.

This study achieved meaningful results, showing that Apc001OA and Apc001OA‐d6, which specifically target sclerostin loop3, can promote BMD, improve bone strength and enhance muscle properties without causing cardiovascular adverse events in ORX mice. Apc001OA and Apc001OA‐d6 could be potential agents for improving bone and muscle health in male patients with osteoporosis. However, this study has some limitations. We determined the dosage of the aptamers based on previous studies [16, 17, 18], and we did not investigate the dose–response relationship of aptamers. We did not investigate the circulation half‐life of the two aptamers in ORX mice. We did not observe the long‐term effects of the Apc001OA and Apc001OA‐d6. Furthermore, we only observed the histological sections of the heart but did not evaluate the cardiovascular functions. And deep‐going studies focusing on the crosstalk between muscle and bone were needed to be conducted.

In conclusion, we confirmed for the first time that novel aptamers targeting sclerostin loop3, Apc001OA and Apc001OA‐d6, can significantly improve BMD, bone microarchitecture, skeletal mechanical properties, muscle function and the histological properties of both muscles and bones, without increasing cardiovascular adverse events in ORX mice. Apc001OA and Apc001OA‐d6 have the potential to treat male osteoporosis and are worthy of further clinical research.

## Ethics Statement

All animal research procedures were approved by the Animal Welfare & Ethics Committee of PUMCH (No. for the application: XHDW‐2022‐068). All sections of the present study adhere to the ‘ARRIVE guidelines’ for reporting in vivo experiments in animal research.

## Conflicts of Interest

The authors declare no conflicts of interest.

## Supporting information


**Figure S1** Structure diagram of the two aptamers Apc001OA and Apc001OA‐d6. (a) Structure diagram of the aptamer Apc001OA. (b) Structure diagram of the aptamer Apc001OA‐d6. The structure diagram was obtained from the Law Sau Fai Institute for Advancing Translational Medicine in Bone and Joint Diseases (TMBJ), School of Chinese Medicine, Hong Kong Baptist University.
**Figure S2** Other characteristics of trabecular bone microarchitecture properties of mice after 12 weeks of treatment. Tb.BS/TV: trabecular bone surface area per total volume, Tb.Conn.D: trabecular connectivity density, Tb.BS/BV: trabecular bone surface area per bone volume, Tb.SMI: trabecular structure model index, SHAM: sham, PBS: phosphate‐buffered saline, Apc001OA and APC001OA‐d6 represent the two groups treated with the two sclerostin aptamers, ALN: alendronate, PTH 1–34: teriparatide.

**Table S1** Primer sequence information related to multiple gene expression of WNT pathway in qPCR.

## Data Availability

All data generated or analysed during this study are not publicly available but are available from the corresponding author at reasonable request.

## References

[1] K. E. Ensrud and C. J. Crandall , “Osteoporosis,” Annals of Internal Medicine 167 (2017): Itc17–itc32. 28761958 10.7326/AITC201708010

[2] T. Vilaca , R. Eastell , and M. Schini , “Osteoporosis in Men,” Lancet Diabetes & Endocrinology 10 (2022): 273–283. 35247315 10.1016/S2213-8587(22)00012-2

[3] C. W. Sing , T. C. Lin , S. Bartholomew , et al., “Global Epidemiology of Hip Fractures: Secular Trends in Incidence Rate, Post‐Fracture Treatment, and All‐Cause Mortality,” Journal of Bone and Mineral Research: The Official Journal of the American Society for Bone and Mineral Research 38 (2023): 1064–1075. 37118993 10.1002/jbmr.4821

[4] R. Bajracharya , J. M. Guralnik , M. D. Shardell , et al., “Long‐Term Sex Differences in All‐Cause and Infection‐Specific Mortality Post Hip Fracture,” Journal of the American Geriatrics Society 70 (2022): 2107–2114. 35415882 10.1111/jgs.17800 PMC9283265

[5] F. Marini , F. Giusti , G. Palmini , and M. L. Brandi , “Role of Wnt Signaling and Sclerostin in Bone and as Therapeutic Targets in Skeletal Disorders,” Osteoporosis International 34 (2023): 213–238. 35982318 10.1007/s00198-022-06523-7

[6] F. Cosman , D. B. Crittenden , J. D. Adachi , et al., “Romosozumab Treatment in Postmenopausal Women With Osteoporosis,” New England Journal of Medicine 375 (2016): 1532–1543. 27641143 10.1056/NEJMoa1607948

[7] A. Miyauchi , E. Hamaya , J. Shimauchi , Y. Yoshinaga , and K. Nishi , “Effectiveness of Romosozumab in Patients With Osteoporosis at High Fracture Risk: A Japanese Real‐World Study,” Journal of Bone and Mineral Metabolism 42 (2024): 77–89. 38086988 10.1007/s00774-023-01477-0

[8] A. Vestergaard Kvist , J. Faruque , E. Vallejo‐Yagüe , S. Weiler , E. M. Winter , and A. M. Burden , “Cardiovascular Safety Profile of Romosozumab: A Pharmacovigilance Analysis of the US Food and Drug Administration Adverse Event Reporting System (FAERS),” Journal of Clinical Medicine 10 (2021): 1660. 33924496 10.3390/jcm10081660 PMC8070537

[9] Z. Zhao , K. Yan , Q. Guan , Q. Guo , and C. Zhao , “Mechanism and Physical Activities in Bone‐Skeletal Muscle Crosstalk,” Frontiers in Endocrinology 14 (2023): 1287972. 38239981 10.3389/fendo.2023.1287972 PMC10795164

[10] E. Gielen , J. Dupont , M. Dejaeger , and M. R. Laurent , “Sarcopenia, Osteoporosis and Frailty,” Metabolism, Clinical and Experimental 145 (2023): 155638. 37348597 10.1016/j.metabol.2023.155638

[11] S. Zhou , H. Si , L. Wu , et al., “Association Between Handgrip Strength Weakness and Asymmetry With Incident Hip Fracture Among Older Chinese Adults,” Archives of Gerontology and Geriatrics 122 (2024): 105385. 38417298 10.1016/j.archger.2024.105385

[12] A. Gandham , G. Gregori , L. Johansson , et al., “Sarcopenia Definitions and Their Association With Fracture Risk in Older Swedish Women,” Journal of Bone and Mineral Research: The Official Journal of the American Society for Bone and Mineral Research 39 (2024): 453–461. 38477811 10.1093/jbmr/zjae026 PMC11262149

[13] I. Aryana , S. S. Rini , and C. H. Soejono , “The Importance of Sclerostin as Bone‐Muscle Mediator Crosstalk,” Annals of Geriatric Medicine and Research 26 (2022): 72–82. 35599457 10.4235/agmr.22.0036 PMC9271392

[14] A. Suzuki , R. Minamide , and J. Iwata , “WNT/β‐Catenin Signaling Plays a Crucial Role in Myoblast Fusion Through Regulation of Nephrin Expression During Development,” Development 145 (2018): dev168351. 30389854 10.1242/dev.168351 PMC6288386

[15] L. Li , S. Xu , H. Yan , et al., “Nucleic Acid Aptamers for Molecular Diagnostics and Therapeutics: Advances and Perspectives,” Angewandte Chemie (International Ed. in English) 60 (2021): 2221–2231. 32282107 10.1002/anie.202003563

[16] Y. Yu , L. Wang , S. Ni , et al., “Targeting Loop3 of Sclerostin Preserves Its Cardiovascular Protective Action and Promotes Bone Formation,” Nature Communications 13 (2022): 4241. 10.1038/s41467-022-31997-8 PMC9307627 35869074

[17] L. Wang , Y. Yu , S. Ni , et al., “Therapeutic Aptamer Targeting Sclerostin Loop3 for Promoting Bone Formation Without Increasing Cardiovascular Risk in Osteogenesis Imperfecta Mice,” Theranostics 12 (2022): 5645–5674. 35966595 10.7150/thno.63177 PMC9373813

[18] H. Zhang , S. Yu , S. Ni , et al., “A Bimolecular Modification Strategy for Developing Long‐Lasting Bone Anabolic Aptamer,” Molecular Therapy—Nucleic Acids 34 (2023): 102073. 38074899 10.1016/j.omtn.2023.102073 PMC10709176

[19] G. Miyamura , H. Wakabayashi , N. Nagao , et al., “Prevention of Bone Loss and Improvement of Pain‐Related Behavior in Hind Limb‐Unloaded Mice by Administration of Teriparatide and Bisphosphonate,” Modern Rheumatology 31 (2021): 733–742. 32646253 10.1080/14397595.2020.1782592

[20] A. Gubu , Y. Ma , S. Yu , et al., “Unique Quinoline Orientations Shape the Modified Aptamer to Sclerostin for Enhanced Binding Affinity and Bone Anabolic Potential,” Molecular Therapy—Nucleic Acids 35, no. 1 (2024): 102146. 38444701 10.1016/j.omtn.2024.102146 PMC10914587

[21] J. Hu , B. Zhou , X. Lin , et al., “Impaired Bone Strength and Bone Microstructure in a Novel Early‐Onset Osteoporotic Rat Model With a Clinically Relevant PLS3 Mutation,” eLife 12 (2023): e80365. 37083757 10.7554/eLife.80365 PMC10159618

[22] L. Liu , C. Peritore , J. Ginsberg , J. Shih , S. Arun , and G. Donmez , “Protective Role of SIRT5 Against Motor Deficit and Dopaminergic Degeneration in MPTP‐Induced Mice Model of Parkinson's Disease,” Behavioural Brain Research 281 (2015): 215–221. 25541039 10.1016/j.bbr.2014.12.035

[23] Y. Zhang , D. Ogbu , S. Garrett , Y. Xia , and J. Sun , “Aberrant Enteric Neuromuscular System and Dysbiosis in Amyotrophic Lateral Sclerosis,” Gut Microbes 13 (2021): 1996848. 34812107 10.1080/19490976.2021.1996848 PMC8632307

[24] D. W. Dempster , J. E. Compston , M. K. Drezner , et al., “Standardized Nomenclature, Symbols, and Units for Bone Histomorphometry: A 2012 Update of the Report of the ASBMR Histomorphometry Nomenclature Committee,” Journal of Bone and Mineral Research: The Official Journal of the American Society for Bone and Mineral Research 28 (2013): 2–17. 23197339 10.1002/jbmr.1805 PMC3672237

[25] S. Yu , D. Li , N. Zhang , et al., “Drug Discovery of Sclerostin Inhibitors,” Acta Pharmaceutica Sinica B 12 (2022): 2150–2170. 35646527 10.1016/j.apsb.2022.01.012 PMC9136615

[26] E. S. Vasiliadis , D. S. Evangelopoulos , A. Kaspiris , I. S. Benetos , C. Vlachos , and S. G. Pneumaticos , “The Role of Sclerostin in Bone Diseases,” Journal of Clinical Medicine 11 (2022): 806. 35160258 10.3390/jcm11030806 PMC8836457

[27] M. R. McClung , A. Grauer , S. Boonen , et al., “Romosozumab in Postmenopausal Women With Low Bone Mineral Density,” New England Journal of Medicine 370 (2014): 412–420. 24382002 10.1056/NEJMoa1305224

[28] K. G. Saag , J. Petersen , M. L. Brandi , et al., “Romosozumab or Alendronate for Fracture Prevention in Women With Osteoporosis,” New England Journal of Medicine 377 (2017): 1417–1427. 28892457 10.1056/NEJMoa1708322

[29] E. M. Lewiecki , T. Blicharski , S. Goemaere , et al., “A Phase III Randomized Placebo‐Controlled Trial to Evaluate Efficacy and Safety of Romosozumab in Men With Osteoporosis,” Journal of Clinical Endocrinology and Metabolism 103 (2018): 3183–3193. 29931216 10.1210/jc.2017-02163

[30] M. C. DeRosa , A. Lin , P. Mallikaratchy , et al., “In Vitro Selection of Aptamers and Their Applications,” Nature Reviews Methods Primers 3 (2023): 55. 10.1038/s43586-023-00247-6 PMC10647184 37969927

[31] C. C. Lee , C. M. Hung , C. H. Chen , et al., “Novel Aptamer‐Based Small‐Molecule Drug Screening Assay to Identify Potential Sclerostin Inhibitors Against Osteoporosis,” International Journal of Molecular Sciences 22 (2021): 8320. 34361085 10.3390/ijms22158320 PMC8348959

[32] R. Iwamoto , M. Koide , N. Udagawa , and Y. Kobayashi , “Positive and Negative Regulators of Sclerostin Expression,” International Journal of Molecular Sciences 23 (2022): 4895. 35563281 10.3390/ijms23094895 PMC9102037

[33] R. Sheng , M. Cao , M. Song , et al., “Muscle‐Bone Crosstalk via Endocrine Signals and Potential Targets for Osteosarcopenia‐Related Fracture,” Journal of Orthopaedic Translation 43 (2023): 36–46. 38021216 10.1016/j.jot.2023.09.007 PMC10654153

[34] G. Li , L. Zhang , D. Wang , et al., “Muscle‐Bone Crosstalk and Potential Therapies for Sarco‐Osteoporosis,” Journal of Cellular Biochemistry 120 (2019): 14262–14273. 31106446 10.1002/jcb.28946 PMC7331460

[35] M. C. K. Severinsen and B. K. Pedersen , “Muscle‐Organ Crosstalk: The Emerging Roles of Myokines,” Endocrine Reviews 41 (2020): 594–609. 32393961 10.1210/endrev/bnaa016 PMC7288608

[36] L. Möckel , M. Bartneck , and C. Möckel , “Risk of Falls in Postmenopausal Women Treated With Romosozumab: Preliminary Indices From a Meta‐Analysis of Randomized, Controlled Trials,” Osteoporosis and Sarcopenia 6 (2020): 20–26. 32226829 10.1016/j.afos.2020.02.003 PMC7093685

[37] P. Cisternas , J. P. Henriquez , E. Brandan , and N. C. Inestrosa , “Wnt Signaling in Skeletal Muscle Dynamics: Myogenesis, Neuromuscular Synapse and Fibrosis,” Molecular Neurobiology 49 (2014): 574–589. 24014138 10.1007/s12035-013-8540-5

