# Supplementary material for: Novel Aptamers Targeting Sclerostin Loop3 Improve Skeletal and Muscle Properties Without Adverse Cardiovascular Effects in Orchiectomized Mice
Source: J Cachexia Sarcopenia Muscle. 2025 Jun 4;16(3):e13831. doi: 10.1002/jcsm.13831 (PMC12134771; doi:10.1002/jcsm.13831)
Supplement: Supplementary file 1 — Figure S1 Structure diagram of the two aptamers Apc001OA and Apc001OA‐d6. (a) Structure diagram of the aptamer Apc001OA. (b) Structure diagram of the aptamer Apc001OA‐d6. The structure diagram was obtained from the Law Sau Fai Institute for Advancing Translational Medicine in Bone and Joint Diseases (TMBJ), School of Chinese Medicine, Hong Kong Baptist University. Figure S2 Other characteristics of trabecular bone microarchitecture properties of mice after 12 weeks of treatment. Tb.BS/TV: trabecular bone surface area per total volume, Tb.Conn.D: trabecular connectivity density, Tb.BS/BV: trabecular bone surface area per bone volume, Tb.SMI: trabecular structure model index, SHAM: sham, PBS: phosphate‐buffered saline, Apc001OA and APC001OA‐d6 represent the two groups treated with the two sclerostin aptamers, ALN: alendronate, PTH 1–34: teriparatide. [file JCSM-16-e13831-s002.pptx]

## Slide 1
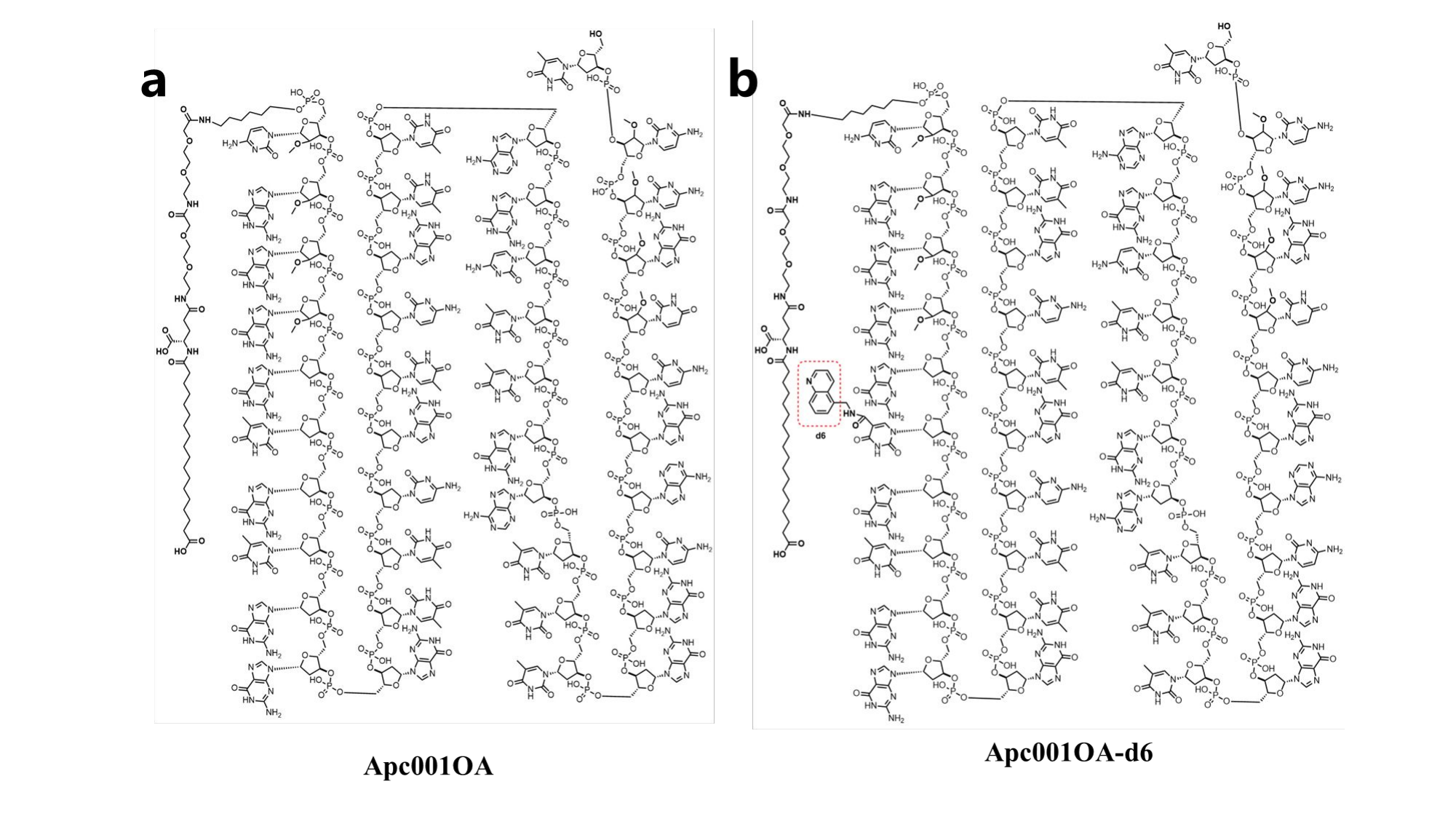

## Slide 2
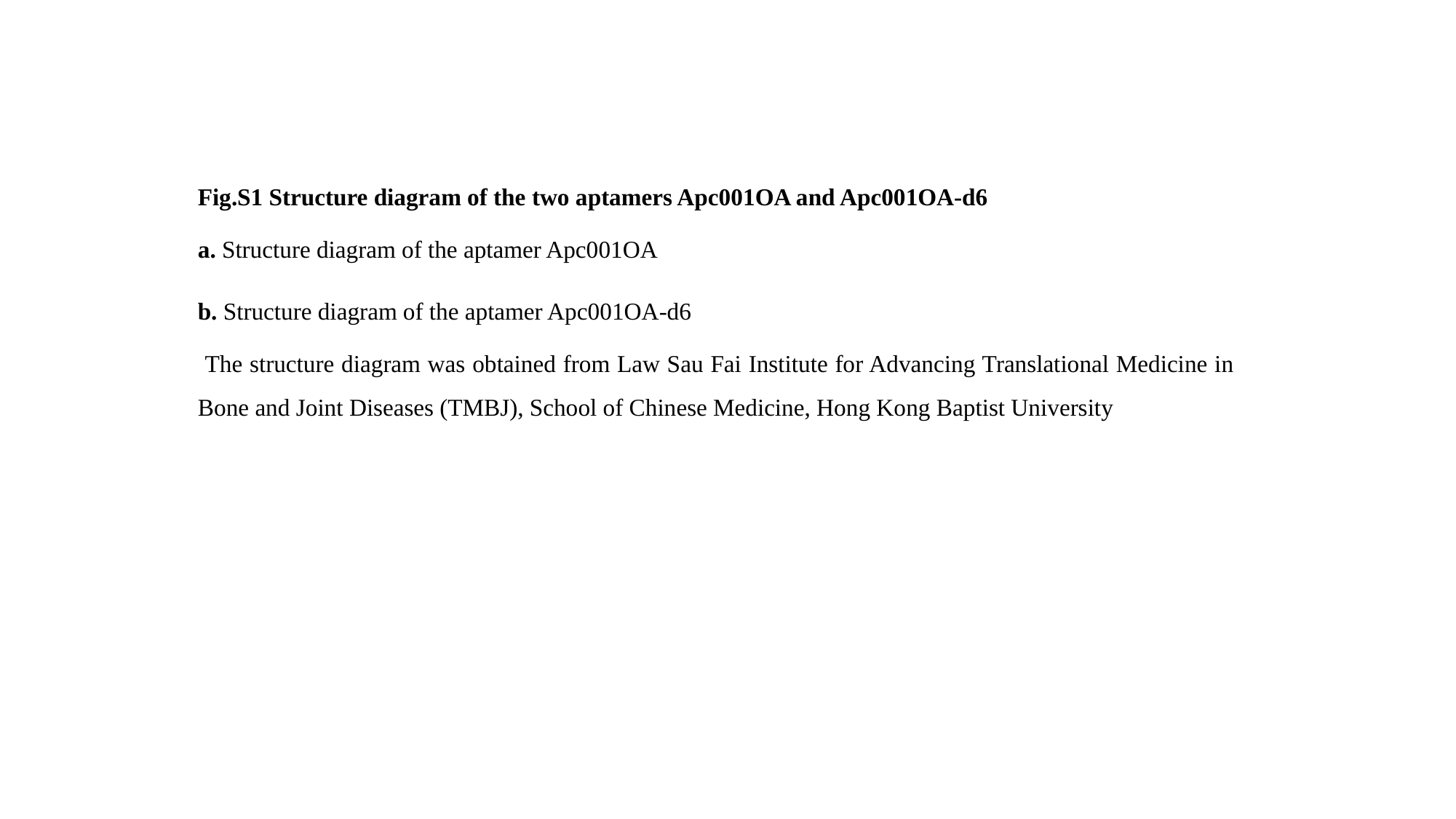

Fig.S1 Structure diagram of the two aptamers Apc001OA and Apc001OA-d6
a. Structure diagram of the aptamer Apc001OA
b. Structure diagram of the aptamer Apc001OA-d6
 The structure diagram was obtained from Law Sau Fai Institute for Advancing Translational Medicine in Bone and Joint Diseases (TMBJ), School of Chinese Medicine, Hong Kong Baptist University

## Slide 3
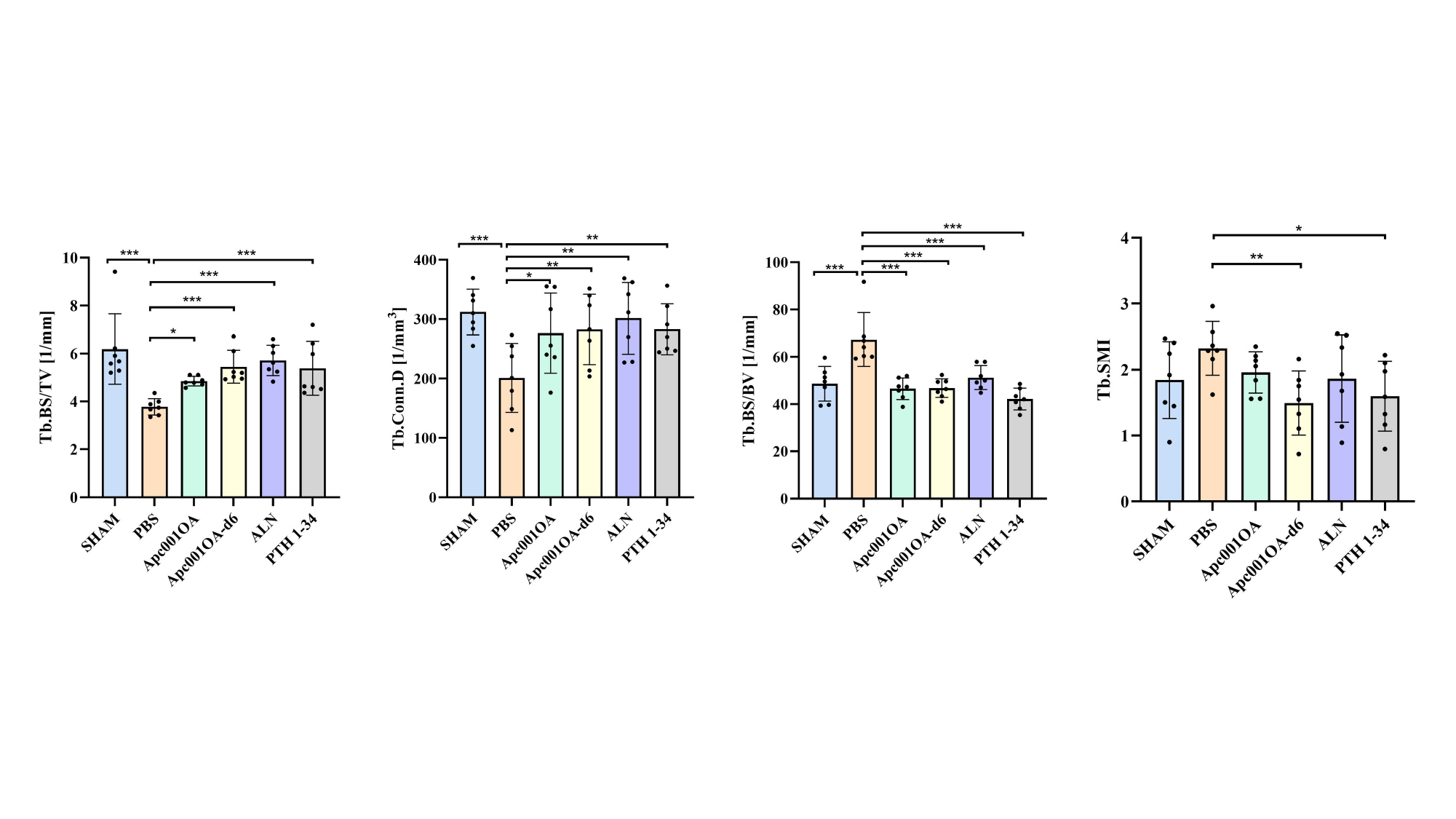

## Slide 4
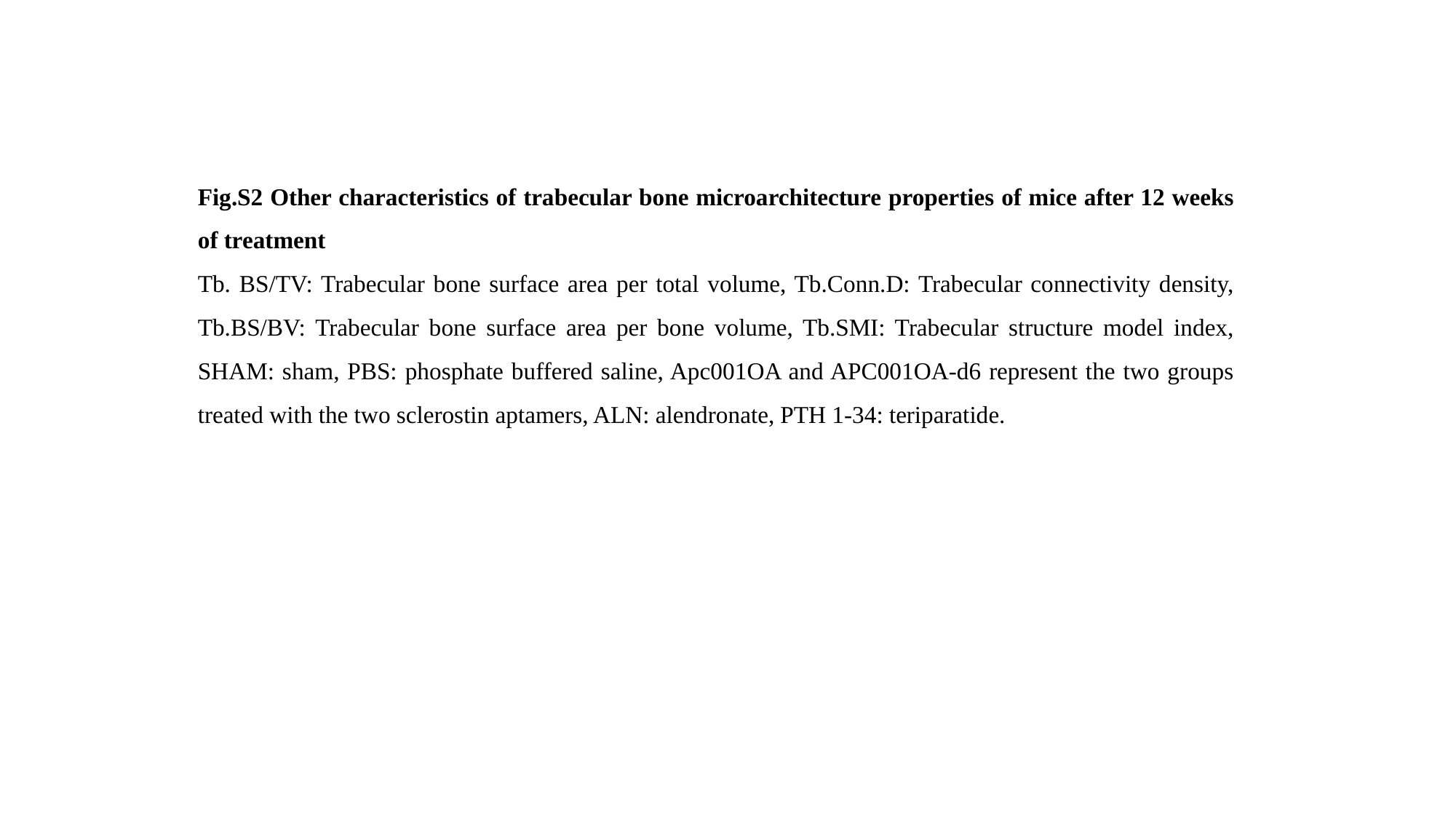

Fig.S2 Other characteristics of trabecular bone microarchitecture properties of mice after 12 weeks of treatment
Tb. BS/TV: Trabecular bone surface area per total volume, Tb.Conn.D: Trabecular connectivity density, Tb.BS/BV: Trabecular bone surface area per bone volume, Tb.SMI: Trabecular structure model index, SHAM: sham, PBS: phosphate buffered saline, Apc001OA and APC001OA-d6 represent the two groups treated with the two sclerostin aptamers, ALN: alendronate, PTH 1-34: teriparatide.

## Slide 5
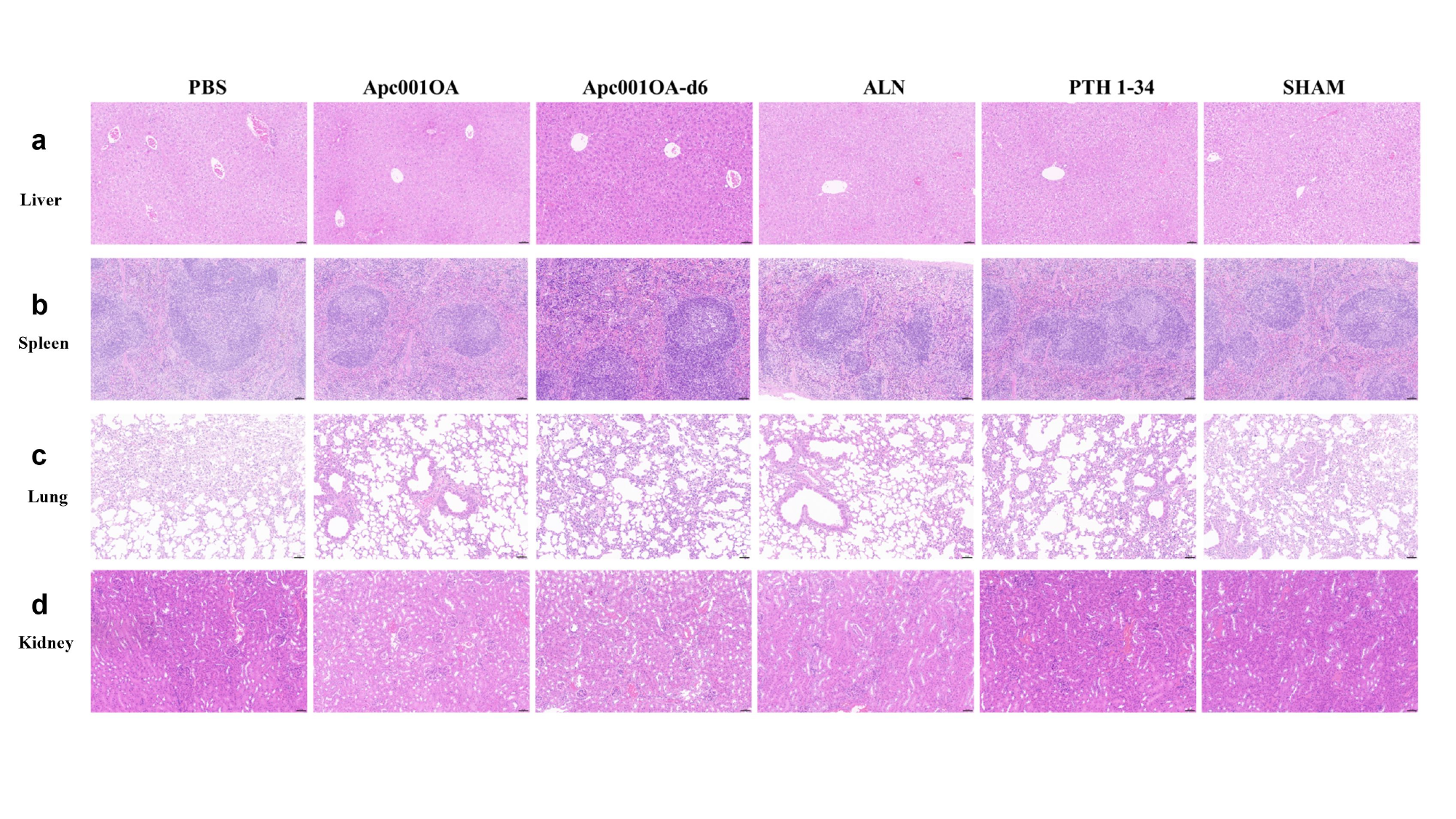

## Slide 6
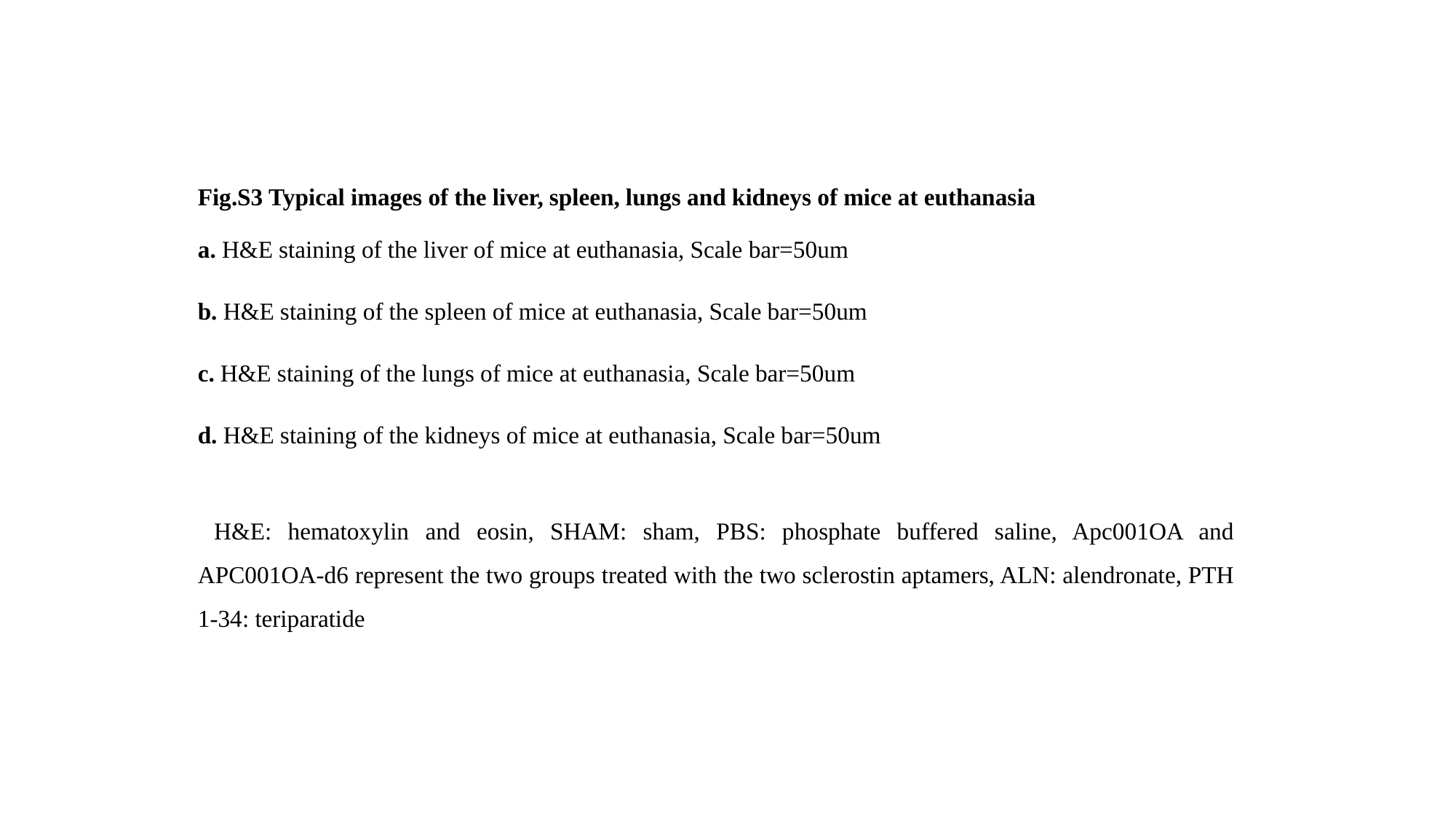

Fig.S3 Typical images of the liver, spleen, lungs and kidneys of mice at euthanasia
a. H&E staining of the liver of mice at euthanasia, Scale bar=50um
b. H&E staining of the spleen of mice at euthanasia, Scale bar=50um
c. H&E staining of the lungs of mice at euthanasia, Scale bar=50um
d. H&E staining of the kidneys of mice at euthanasia, Scale bar=50um
 H&E: hematoxylin and eosin, SHAM: sham, PBS: phosphate buffered saline, Apc001OA and APC001OA-d6 represent the two groups treated with the two sclerostin aptamers, ALN: alendronate, PTH 1-34: teriparatide
