# Supplementary material for: Novel Aptamers Targeting Sclerostin Loop3 Improve Skeletal and Muscle Properties Without Adverse Cardiovascular Effects in Orchiectomized Mice
Source: J Cachexia Sarcopenia Muscle. 2025 Jun 4;16(3):e13831. doi: 10.1002/jcsm.13831 (PMC12134771; doi:10.1002/jcsm.13831)
Supplement: Supplementary file 2 — Table S1 Primer sequence information related to multiple gene expression of WNT pathway in qPCR. [file JCSM-16-e13831-s001.docx]

| **Table S1.** Primer sequence information related to multiple gene expression of WNT pathway in qPCR | |
| --- | --- |
| Subjects | Sequence information |
| Sost-F | 5’ - AGCCTTCAGGAATGATGCCAC - 3’ |
| Sost-R | 5’ - CTTTGGCGTCATAGGGATGGT - 3’ |
| Lrp5-F | 5’ – AAGGGTGCTGTGTACTGGAC - 3’ |
| Lrp5-R | 5’ - AGAAGAGAACCTTACGGGACG- 3’ |
| Axin1-F | 5’ – CTCCAAGCAGAGGACAAAATCA - 3’ |
| Axin1-R | 5’ – GGATGGGTTCCCCACAGAAATA - 3’ |
| Ctnnb1-F | 5’ – ATGGAGCCGGACAGAAAAGC - 3’ |
| Ctnnb1-R | 5’ – CTTGCCACTCAGGGAAGGA - 3’ |
| Lef1-F | 5’ - TGTTTATCCCATCACGGGTGG - 3’ |
| Lef1-R | 5’ - CATGGAAGTGTCGCCTGACAG - 3’ |
| Bglap-F | 5’ - CTCACAGATGCCAAGCCCA - 3’ |
| Bglap-R | 5’ - CCAAGGTAGCGCCGGAGTCT - 3’ |
| Runx2-F | 5’ – AGAGTCAGATTACAGATCCCAGG - 3’ |
| Runx2-R | 5’ – TGGCTCTTCTTACTGAGAGAGG - 3’ |
| Sp7-F | 5’ - ATGGCGTCCTCTCTGCTTG - 3’ |
| Sp7-R | 5’ - TGAAAGGTCAGCGTATGGCTT- 3’ |
| Gapdh-F | 5’ - AGGTCGGTGTGAACGGATTTG - 3’ |
| Gapdh-R | 5’ - TGTAGACCATGTAGTTGAGGTCA- 3’ |
| Abbreviations: Sost: sclerostin; Lrp5: low density lipoprotein receptor-related protein 5; Axin1:axin1; Ctnnb1: catenin beta 1; Lef1: lymphoid enhancer binding factor 1; Bglap: bone gamma carboxyglutamate protein; Runx2: runt related transcription factor 2; Sp7: Sp7 transcription factor 7; Gapdh: glyceraldehyde-3-phosphate dehydrogenase | |
